# Transcriptome profiling of human hypothalamic agouti-related protein and proopiomelanocortin neurons regulating energy homeostasis

**DOI:** 10.1038/s41467-026-76688-w

**Published:** 2026-08-15

**Authors:** Szabolcs Takács, Katalin Skrapits, Balázs Göcz, Éva Rumpler, Miklós Sárvári, Barbara Göblyös, Dalma Biri, Szilárd Póliska, Gergely Rácz, András Matolcsy, Erik Hrabovszky

**Affiliations:** 1 Laboratory of Reproductive Neurobiology, Hun-Ren Institute of Experimental Medicine, Budapest, Hungary; 2 Roska Tamás Doctoral School of Sciences and Technology, Faculty of Information Technology and Bionics, Pázmány Péter Catholic University, Budapest, Hungary; 3 Department of Biochemistry and Molecular Biology, Faculty of Medicine, University of Debrecen, Debrecen, Hungary; 4 Department of Pathology and Experimental Cancer Research, Semmelweis University, Budapest, Hungary

**Keywords:** Molecular neuroscience, Hypothalamus

## Abstract

We present and validate a pioneering ‘IHC/LCM-Seq’ method for transcriptome profiling of spatially defined neuronal cell types detected with immunohistochemistry in sections of formaldehyde-fixed human brains. Application of IHC/LCM-Seq to male human hypothalami enables the identification of 14,000–16,000 transcripts in agouti-related protein (AgRP) neurons, which drive appetite and energy storage, and proopiomelanocortin (POMC) neurons, which suppress feeding and promote energy expenditure. These cell types differ from each other, and from fertility-regulating kisspeptin neurons, in their distinct enrichments of co-transmitters, transcription factors, regulatory (lnc) RNAs and receptors. The AgRP neuron transcriptome is rich in receptors for proinflammatory cytokines, metabolic hormones and growth hormone, whereas POMC neurons express reproductive hormone-, glucagon-like peptide-, calcitonin-, and endocannabinoid receptors. IHC/LCM-Seq, a versatile spatial transcriptomic approach for characterizing cell types in *postmortem* human brain tissue, opens a new window onto the molecular mechanisms that regulate energy homeostasis and highlights potential pharmacological targets for weight-management strategies.

## Abstract (web summary)

Gene expression profiling of diverse cell types from postmortem human brains is challenging. Here, the authors present a versatile spatial transcriptomic approach to uncover the molecular signatures of appetite-regulating AgRP and POMC neurons.

## Introduction

The hypothalamic arcuate nucleus, also known in humans as the infundibular nucleus (INF), plays a central role in energy homeostasis, primarily through the actions of two functionally antagonistic neuronal cell types: agouti-related protein (AgRP) neurons, which stimulate appetite and promote energy conservation, and proopiomelanocortin (POMC) neurons, which suppress food intake and enhance energy expenditure^1^. AgRP and POMC neurons are highly responsive to peripheral metabolic cues—including leptin, insulin, and ghrelin—and their dysfunction is strongly linked to disorders of energy homeostasis, such as obesity, anorexia, and diabetes^1,2^. Defining the molecular signatures of AgRP and POMC neurons is therefore fundamental for identifying therapeutic strategies for metabolic disorders. In particular, detailed characterization of their receptor profiles may reveal previously unexplored mechanisms regulating energy homeostasis and guide targeted approaches to obesity treatment. For example, expression of *GLP1R* in anorexigenic POMC neurons^3^ likely contributes to the anti-obesity effects of semaglutide. Similarly, semaglutide combined with cagrilintide^4^, a long-acting dual amylin/calcitonin receptor agonist, may benefit from *CALCR* expression in this cell type, provided that POMC neurons also express the relevant *RAMPs* required for canonical amylin receptor signaling^3^.

Current knowledge of the regulatory mechanisms within hypothalamic neuronal circuits targeted by obesity and diabetes treatments has largely been derived from animal studies, limiting the translational potential for developing effective therapies in humans. Recent advances in single-nucleus RNA sequencing (snRNA-seq) have enabled the creation of a spatiocellular transcriptional map (“HYPOMAP”) of the human hypothalamus^3^. The authors identified ~2000 transcripts per cell and integrated their snRNA-seq data with a hypothalamic expression matrix derived from publicly available whole-brain human datasets^5^. Through multi-level hierarchical unsupervised clustering, they delineated 291 hypothalamic neuronal clusters, including a single AgRP neuron cluster defined by 5 signature genes, and three distinct POMC neuron clusters characterized by 8 marker genes^3^.

Despite their high translational value, the sensitivity of various single-cell sequencing techniques is severely limited by the small quantity of input RNA. By contrast, bulk sequencing of neuronal pools isolated via fluorescence-activated cell sorting (FACS) or laser capture microdissection (LCM) enables the detection of rare transcripts (such as a large subset of long noncoding RNAs; lncRNAs) and produces highly detailed transcriptomic profiles that are otherwise difficult to obtain. Our laboratory recently achieved a methodological breakthrough in bulk sequencing by integrating innovative RNA-preserving strategies during immunohistochemistry (IHC) with the isolation of labeled neurons using LCM, followed by RNA-seq^6^. In the present study, we adapted this “IHC/LCM-Seq” approach to human *postmortem* brains fixed by immersion in 4% formaldehyde (FA). This adaptation enabled transcriptomic profiling of AgRP- and POMC-immunoreactive (IR) neurons in the INF, as well as fertility-regulating kisspeptin (KP)-IR neurons^7^ of the same region, thereby allowing comparisons among three distinct neuronal phenotypes. The IHC/LCM-Seq methodology yielded unprecedented transcriptome coverage, identifying ~14,000–16,000 transcripts per cell type. In POMC and AgRP neurons, many showed species-specific expression patterns that differed markedly from those reported in rodents. These included neuropeptides, rare transcription factors, and regulatory RNAs, and, most notably, receptors, some of which may represent promising targets for future weight-management strategies.

## Results

### Immersion-fixation of *postmortem* brains retains RNA integrity

**Fig. 1:**
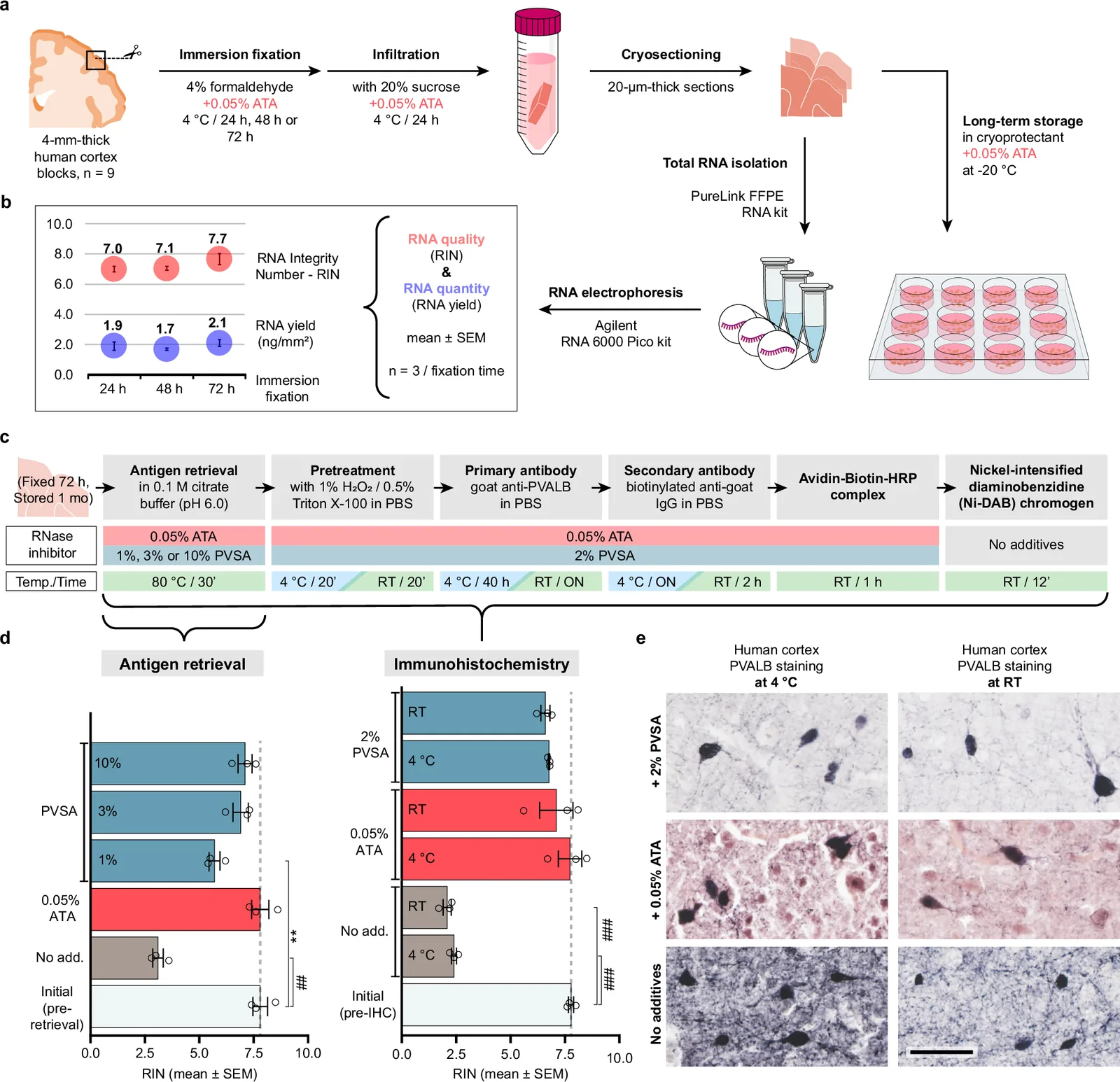
Development and validation of a versatile “IHC/LCM-Seq” method for transcriptome profiling of immunohistochemically labeled human neuronal cell types. **a** Schematic overview of histological procedures assessing the impact of fixation time (24, 48, or 72 h in 4% cold formaldehyde) on RNA integrity. **b** Neocortical tissue samples from a 53-year-old female (Sample #1, Supplementary Data 3) processed in the presence of the RNase inhibitor aluminon (ATA; 0.05%), show comparable RNA quality (RIN > 7.0) and yield across different fixation times (Agilent Bioanalyzer; Supplementary Data 1; *n* = 3/fixation time). **c** Experimental design for optimizing RNA preservation using ATA (0.05%) or polyvinyl sulfonic acid (PVSA; 1-10%) during immunohistochemical detection of cortical parvalbumin (PVALB) neurons. **d** Both ATA and PVSA provide significant protection against degradation during antigen retrieval at 80 °C, while RNA integrity is severely compromised in the absence of RNase inhibitors. ATA (0.05%) and PVSA (2%) also maintained RNA integrity during IHC performed at either room temperature (RT) or 4 °C (Supplementary Data 2; *n* = 3 experimental replicates). **e** Effects of ATA (0.05%) and PVSA (2%) on immunohistochemical signal quality and background. Both additives enable successful detection of PVALB neurons; however, ATA treatment results in increased nonspecific background staining. Scale bar: 50 µm. Statistical annotations: **P < 0.01, ^##^*P* < 0.00001, ^###^*P* < 0.000001 by one-way ANOVA followed by Tukey’s HSD. Exact *P*.adj. values are provided in Supplementary Data 2.

The IHC/LCM-Seq protocol we developed for laboratory rodents began with perfusion fixation of the brain with 4% FA^6^. Because this fixation protocol is usually not applicable to autopsy material, we first assessed how *postmortem* delay and immersion fixation jointly affect tissue RNA integrity. Neocortical blocks (~ 4 mm in each dimension) were dissected from the brain of a 53-year-old woman ~20 h after death and immersion-fixed in cold (4 °C) 4% FA for 24, 48, or 72 h, then infiltrated with 20% sucrose for 24 h. Twenty-µm-thick free-floating sections were prepared using a freezing microtome and stored at −20 °C in a cryoprotectant solution modified for long-term RNA preservation^6^ (Fig. 1a). As we proposed recently^6^, all solutions were supplemented with the potent RNase inhibitor aluminon^8^ (aurintricarboxylic acid, ammonium salt; ATA; 0.05%). Bioanalyzer measurements of total RNA extracted from test sections revealed consistently high RNA integrity (RIN > 7.0) and comparable yields (~ 2 ng/mm^2^ section area), regardless of fixation duration (Fig. 1b and Supplementary Data 1).

### A modified immunohistochemical protocol preserves tissue RNAs

Encouraged by the high RIN values of immersion-fixed samples, we next adapted the IHC/LCM-Seq workflow for use on human tissue sections. The modified immunohistochemical protocol began with epitope retrieval in 0.1 M citrate buffer (pH 6.0, 80 °C, 30 min), supplemented with either 0.05% ATA or polyvinyl sulfonate (PVSA) at 1%, 3%, or 10% (Fig. 1c). In the absence of RNase inhibitors, RIN values dropped sharply from 7.8 ± 0.3 to 3.1 ± 0.2. In contrast, inclusion of 0.05% ATA or PVSA (when used at 3% or 10%) effectively preserved RNA integrity (Fig. 1d and Supplementary Data 2). As a final validation of the RNA-preserving immunohistochemical protocol, we visualized cortical parvalbumin neurons by IHC (Fig. 1e), followed by RNA quality analysis. As expected, sections immunostained without RNase inhibitors showed substantial RNA degradation, with post-IHC RIN values of 2.4 ± 0.1 after IHC at 4 °C and 2.1 ± 0.2 after IHC at room temperature. In contrast, supplementing all antibody and buffer solutions with 0.05% ATA or 2% PVSA effectively preserved RNA, yielding RIN values of 7.7 ± 0.5 (ATA) and 6.8 ± 0.0 (PVSA) at 4 °C, and 7.1 ± 0.8 (ATA) and 6.6 ± 0.2 (PVSA) at RT (Fig. 1d and Supplementary Data 2).

**Fig. 2:**
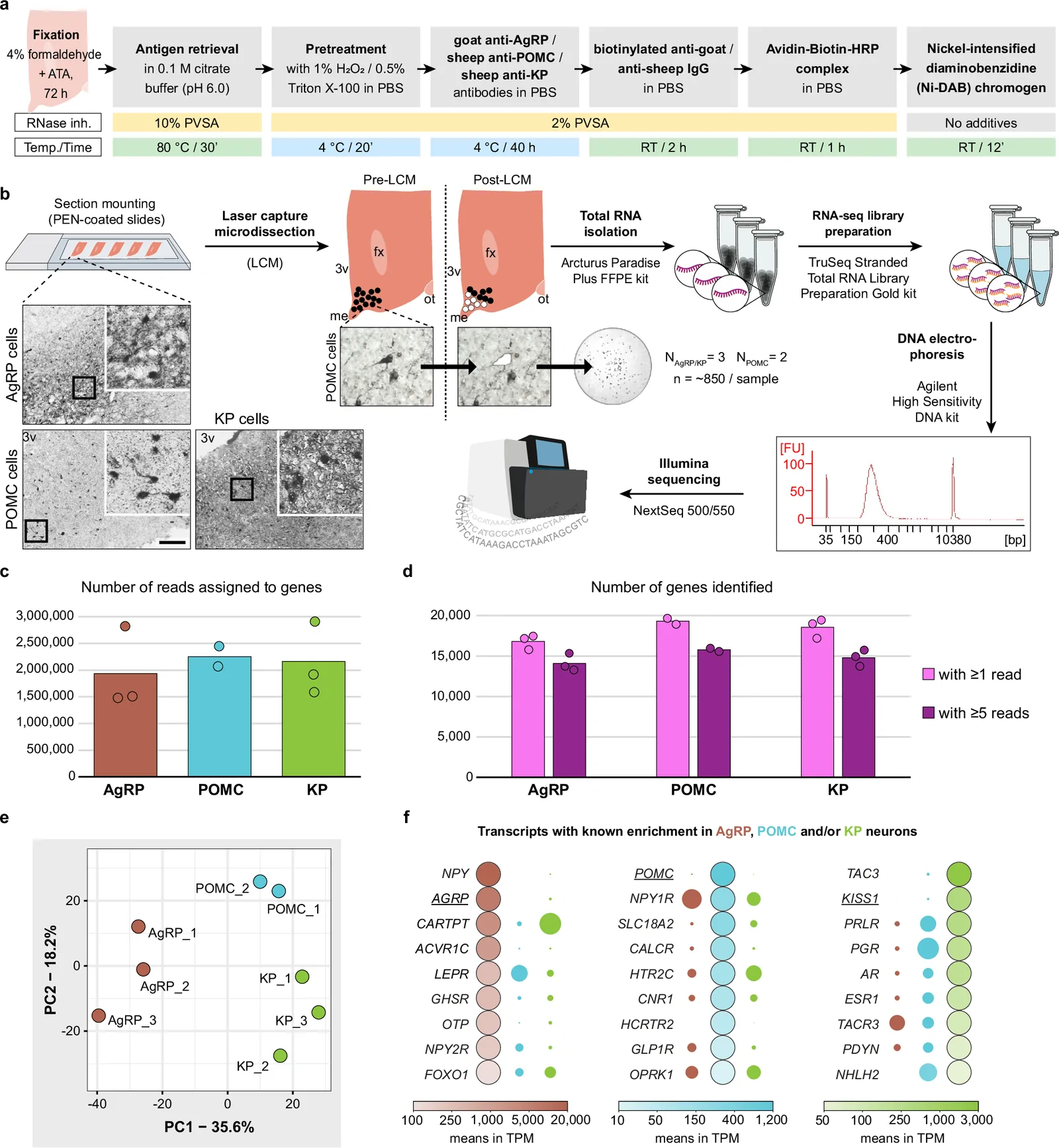
Use of IHC/LCM-Seq for transcriptome profiling of human AgRP, POMC, and KP neurons. **a** Schematic overview and technical details of the RNA-preserving immunohistochemistry (IHC) protocol optimized for transcriptome analysis of human AgRP-, POMC-, and KP-IR neuronal pools. **b** Workflow of the protocol, including laser capture microdissection (LCM), RNA isolation, cDNA library preparation, RNA sequencing, and downstream bioinformatic analyses. Scale bar corresponds to 200 µm in overview images and 50 µm in high-magnification and LCM images. 3v 3rd ventricle, fx fornix, me median eminence, ot optic tract. **c**, **d** Gene-assigned read counts (**c**) and numbers of identified transcripts (**d**) in AgRP (*n* = 3), POMC (*n* = 2), and KP (*n* = 3) neurons at two expression thresholds (Supplementary Data 4). *n* denotes the number of human subjects. **e** Principal component analysis (PCA) showing distinct clustering of the AgRP, POMC, and KP transcriptomes, with PC1 and PC2 explaining ~50% of the total variance (For raw data, see Supplementary Data 5). **f** Representative genes showing cell type-specific enrichment. Color gradients within the large dots indicate transcript abundance as mean TPM (transcripts per million), whereas the smaller dots are proportionally scaled in surface area to reflect lower expression levels.

Of note, ATA tended to compromise specific immunostaining and increase nonspecific background (Fig. 1e), consistent with our previous observations^6^. Therefore, we selected 2% PVSA for inclusion in buffers and antibody solutions in the finalized IHC/LCM-Seq protocol for profiling hypothalamic AgRP, POMC, and KP neurons (Fig. 2a).

### Optimized processing supports RNA sequencing of hypothalamic cells

Pre-IHC RINs from hypothalamic test sections of 8 male subjects used for transcriptomic experiments ranged from 3.1 to 6.7 (Supplementary Data 3). Use of the optimized IHC/LCM-Seq protocol (Fig. 2a) enabled robust immunohistochemical detection of AgRP-, POMC-, and KP-IR neurons in the human mediobasal hypothalamus, followed by their capture with LCM (~ 850 labeled cells per cell type per subject). This was followed by RNA isolation, cDNA library preparation, and Illumina-based RNA sequencing (Fig. 2b). Raw sequencing data have been deposited in BioProject under accession numbers PRJNA1305226 (AgRP- and POMC-IR neuronal phenotypes) and PRJNA1305334 (KP-IR).

Sequencing of eight libraries—comprising 3 AgRP-, 2 POMC-, and 3 KP neuron transcriptomes—generated a total of 211.8 million raw reads (17.6–33 million per sample). Following trimming, adapter removal, alignment to the human reference genome (Ensembl release 111) using STAR (v2.7.11b), and gene assignment with FeatureCounts (subread v2.0.6), an average of 2.2 ± 0.6 million reads per sample (17.6 million total) were uniquely mapped and assigned to genes (Fig. 2c and Supplementary Data 4). This enabled identification of ~18,000 ± 500 transcripts with ≥1 read, and 15,000 ± 400 transcripts with ≥5 reads (mean ± SEM, Fig. 2d and Supplementary Data 4). Principal component analysis (PCA) verified three distinct transcriptomic profiles, each corresponding to a specific cell type (Fig. 2e). The lack of marker neuropeptide transcripts (*AGRP*, *POMC*, and *KISS1*) outside their respective transcriptomes, along with the asymmetric expression of established neuronal phenotype markers, indicated negligible, if any, cross-contamination between transcriptomes (Fig. 2f and Supplementary Data 5).

### Transcription factor profiles differ among neuronal phenotypes

**Fig. 3:**
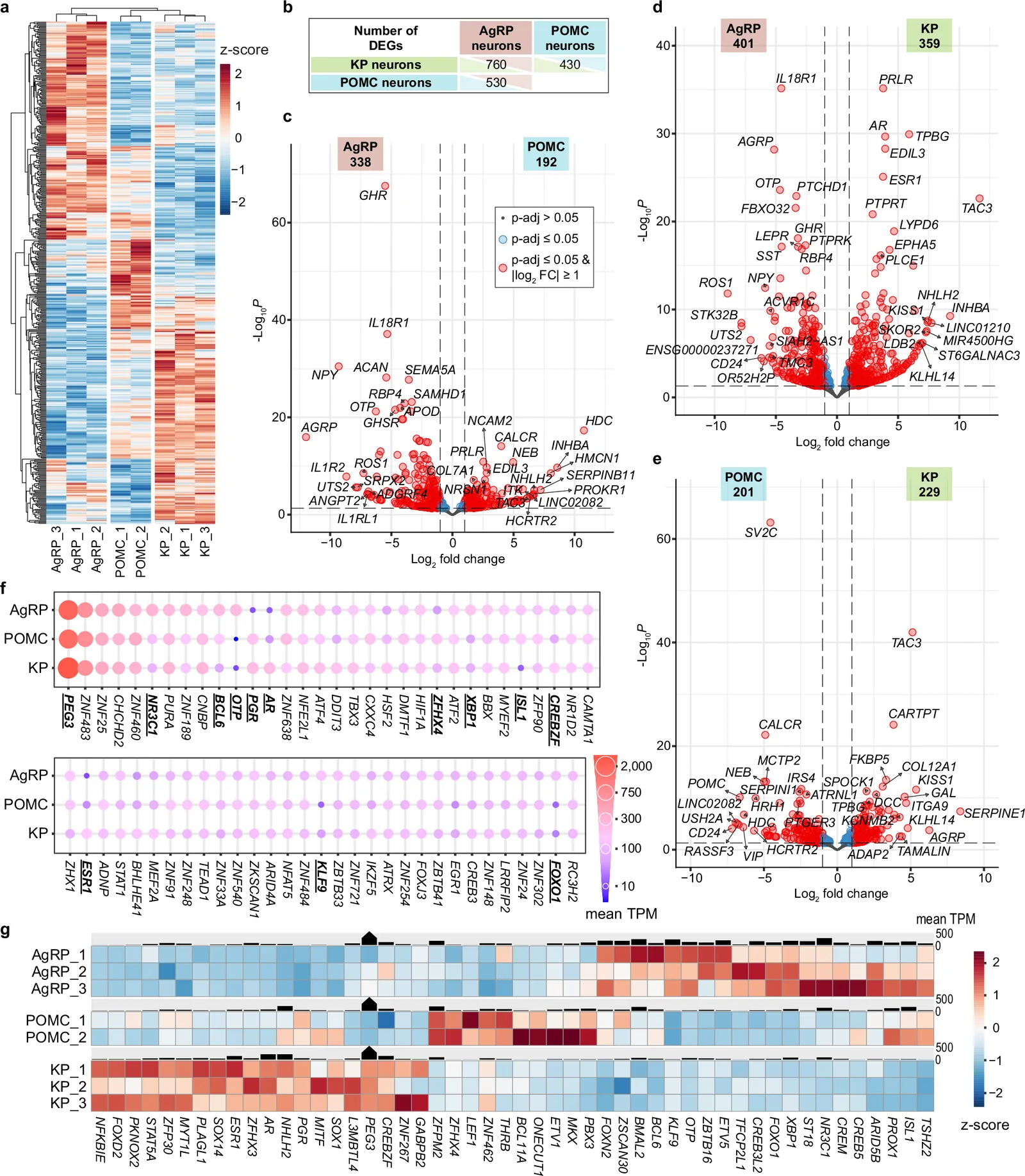
Differential expression of transcripts and transcription factors in human AgRP, POMC, and KP neurons. **a** Heatmap showing the 1,210 transcripts differentially expressed (*P*.adj. <0.05, DESeq2, two-sided Wald test with Benjamini–Hochberg false discovery rate correction) between any two of the AgRP (*n* = 3), POMC (*n* = 2), and KP (*n* = 3) neuronal transcriptomes. *n* denotes the number of human subjects. **b** Table summarizing the number of differentially expressed genes (DEGs) in pairwise comparisons. **c**–**e** Volcano plots showing the relationship between expression magnitude (log_2_ fold change/FC/) and statistical significance (log_10_ *P*.adj.) across all transcripts. Gene symbols denote the top ten upregulated and top ten downregulated transcripts by fold change, as well as the ten transcripts with the lowest adjusted *P* values in each direction. **f** Dot plots of 63 transcription factors (TFs) compiled from the 40 most abundantly expressed TF transcripts in each cell type. Dot size and color are proportional to expression levels (TPM). TFs with significant differential expression (*P*.adj. <0.05) are marked with bold, underlined labels. **g** Heatmap showing cell type-specific expression patterns of 50 TFs differentially expressed between AgRP-, POMC-, and KP neurons (*P*.adj. <0.05). See Supplementary Data 5 for the complete list of expressed genes and Supplementary Data 6 for the complete list of expressed transcription factors, including the exact *P*.adj. values.

A total of 1210 transcripts were significantly enriched in a cell type-specific manner among AgRP, POMC, and KP neurons (adjusted *P* value/*P*.adj./ < 0.05, DESeq2; Supplementary Data 5). Differentially expressed genes (DEGs) are shown as a heatmap (Fig. 3a), numerical expression data (Fig. 3b), and volcano plots across the three pairwise comparisons (Fig. 3c–e). We further examined 902 transcription factor (TF) transcripts annotated in the KEGG BRITE database^9^ to explore their potential roles in shaping the distinct transcriptomic profiles of AgRP, POMC, and KP neurons (Supplementary Data 6). Among the 40 most abundant TFs per cell type (63 in total), 13 were significantly differentially expressed, indicating cell type-specific transcriptional regulation (Fig. 3f and Supplementary Data 6). Expanding the analysis to all expressed TFs revealed 50 transcripts that differed between at least two neuronal phenotypes, underscoring broader cell-type-specific transcriptional regulation (*P*.adj. <0.05, DESeq2; Fig. 3g and Supplementary Data 6). Shared TFs, in turn, may reflect common developmental programs, humoral signals, and afferent inputs. TFs with cell type-specific enrichment (*P*.adj. <0.05, fold change /FC/ > 1.5 *vs*. other cell types) included *NR3C1, OTP, XBP1, KLF9, FOXO1, ETV5, CREM, ZBTB16*, and *ST18* for AgRP neurons; *ZFHX4, ONECUT1*, and *LEF1* for POMC neurons; and *BCL6, ISL1*, and *PROX1* for both AgRP and POMC neurons. Unlike AgRP neurons, POMC cells shared several TFs with KP neurons (*PGR, AR, ESR1, NHLH2*, and* SOX1*). *SOX14, STAT5A*, and *PLAGL1* were enriched in KP neurons (Fig. 3g and Supplementary Data 6).

### AgRP, POMC, and KP neurons express different peptide co-transmitters

**Fig. 4:**
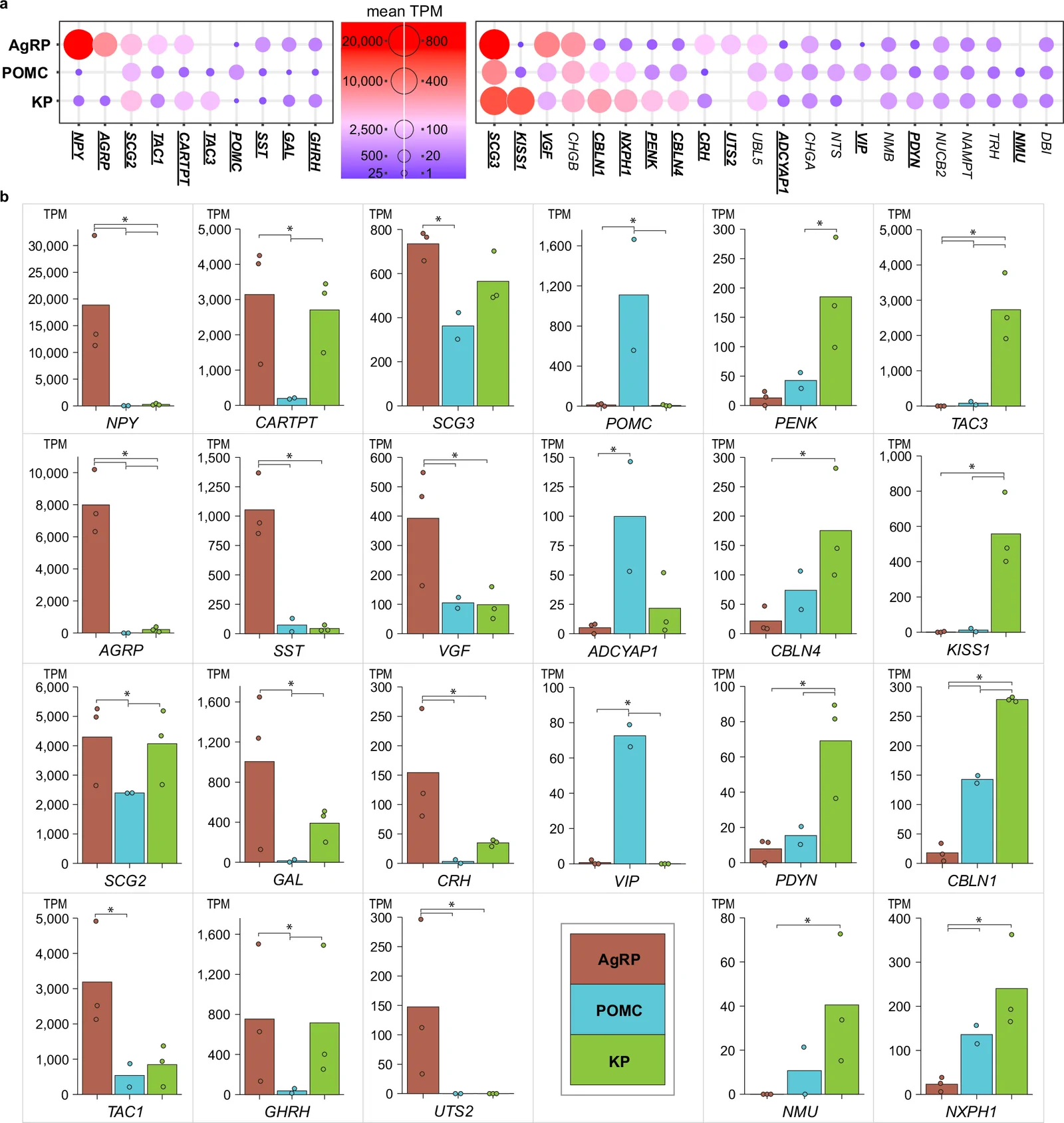
Neuropeptide co-expression in human AgRP-, POMC-, and KP neuronal phenotypes. **a** Dot plots showing the expression of 32 neuropeptide and granin transcripts curated from the Neuropeptide Database (www.neuropeptides.nl), each with a mean expression level ≥40 TPM in at least one of the AgRP (*n* = 3), POMC (*n* = 2), or KP (*n* = 3) neuronal transcriptomes. *n* denotes the number of human subjects. Dot size and color are scaled according to transcript abundance. Bold and underlined symbols indicate statistically significant differences between any two cell types (*P*.adj. <0.05, DESeq2, two-sided Wald test with Benjamini–Hochberg FDR correction). **b** Bar charts showing 23 neuropeptide genes differentially expressed in pairwise comparisons. (*P.adj. <0.05 by DESeq2, two-sided Wald test with Benjamini–Hochberg FDR correction). See Supplementary Data 7 for detailed expression values and statistical results, including the exact *P*.adj. values, for each neuropeptide.

Neuropeptide and granin expression profiles, based on annotations from the Neuropeptide Database^10^ (www.neuropeptides.nl), showed marked differences among the three neuronal phenotypes. Normalized transcript abundances are presented as transcripts per million (TPM) in Fig. 4a, whereas transcripts showing significant differential expression between cell types are shown in Fig. 4b. AgRP neurons were enriched for *NPY*, *AGRP*, *SST*, and *UTS2*, and exhibited higher expression of *TAC1*, *GAL*, *VGF*, and *CRH* compared to POMC and KP neurons. POMC neurons uniquely expressed *POMC* and *VIP*, and had higher *ADCYAP1* transcript levels than AgRP neurons. KP neurons selectively expressed *TAC3* and *KISS1*, and showed elevated expression of *CBLN1*, *NXPH1*, *PENK*, *CBLN4*, *PDYN*, and *NMU* compared to AgRP and POMC neurons (Fig. 4a, b and Supplementary Data 7**)**.

**Fig. 5:**
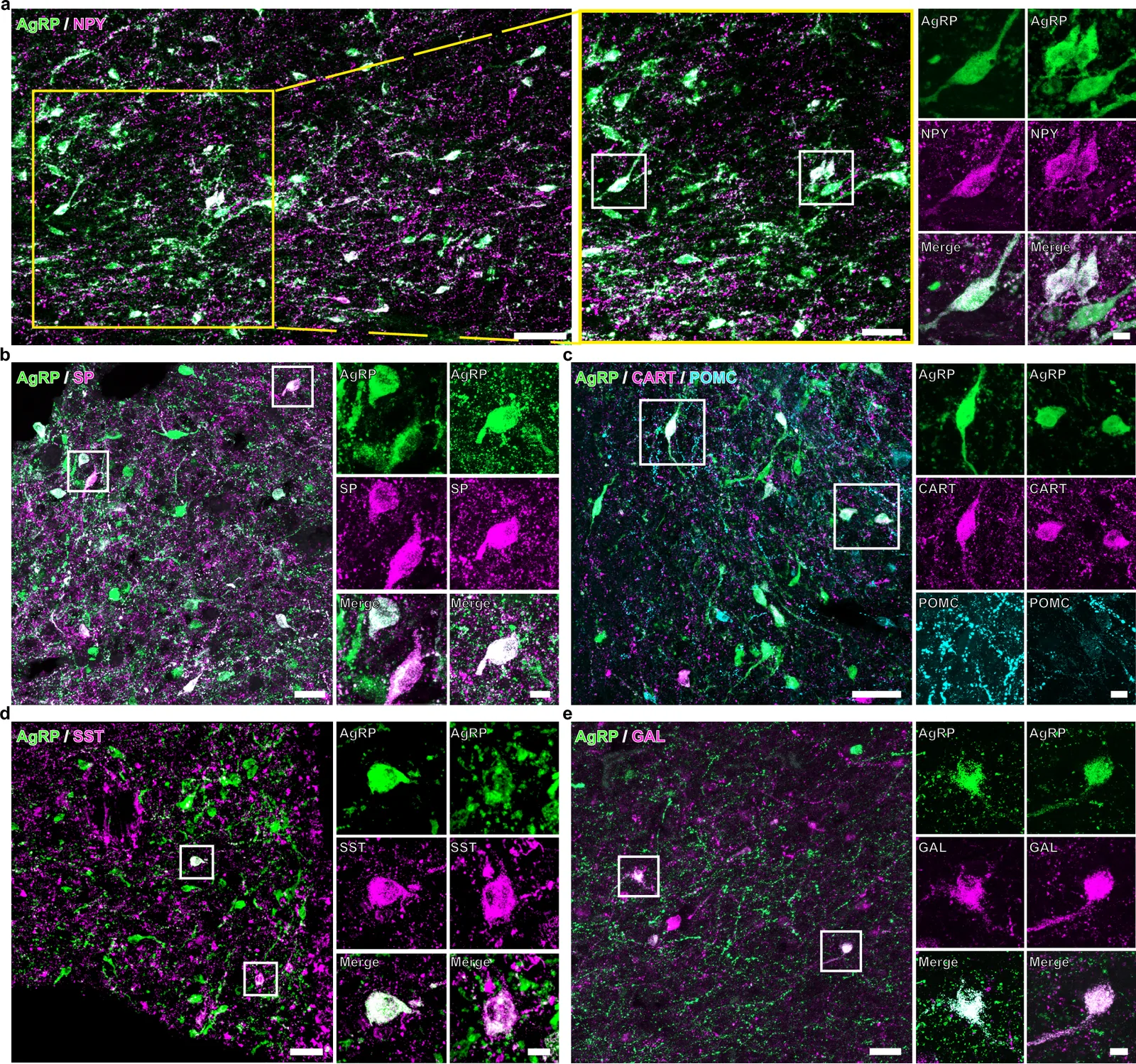
Dual-immunofluorescent validation of neuropeptide co-expression in human AgRP neurons. **a**–**e** Presence of neuropeptide Y (NPY; **a**), substance P (SP; **b**), cocaine- and amphetamine-regulated transcript (CART; **c**), somatostatin (SST; **d**), and galanin (GAL; **e**) labeling (magenta) in varying subsets of AgRP-IR neurons (green). Note that POMC neurons (turquoise in **c**) are devoid of CART immunolabeling. Medium- and high-power images magnified from the boxed areas show examples of dual-labeled neurons in separate and merged channels. Similar results were obtained in two independent experiments, each using two different biological samples. For antibodies, see Supplementary Data 8. Scale bars: 100 µm in low-power image (**a**), 50 µm in the medium-magnification images (**a**–**e**), and 10 µm in the high-power images enlarged from the boxed areas of preceding panels.

Immunofluorescence experiments validating some of these observations confirmed the co-synthesis of neuropeptide Y (encoded by *NPY;* Fig. 5a), substance P (encoded by *TAC1;* Fig. 5b), cocaine- and amphetamine-regulated transcript (CART; encoded by *CARTPT;* Fig. 5c), somatostatin (encoded by *SST*; Fig. 5d), and galanin (encoded by *GAL;* Fig. 5e) in varying subsets of AgRP-IR neurons. Unlike in rodents, human POMC neurons were not IR for CART (Fig. 5c), consistent with earlier reports^11^. For antibodies and reagents, see Supplementary Data 8.

### IHC/LCM-Seq enables sensitive detection of rare lncRNA species

Long noncoding RNAs (lncRNAs) are a diverse class of RNA molecules that play important regulatory roles in gene expression and cellular processes^12^. They represent relatively rare transcript species and are often devoid of a poly(A) tail, making them difficult to capture with conventional sequencing methods. In our study, the combination of random primer-based library preparation with the high sensitivity of the IHC/LCM-Seq method enabled the detection of 1254 medium-to-high abundance lncRNAs (defined by mean TPM ≥ 10 and ≥5 reads in each sample of a given cell type), of which 87 differed significantly (DESeq2) between neuronal phenotypes and 369 showed at least fourfold enrichment (log_2_ FC ≥ 2) in mean TPMs. A complete list of expressed lncRNAs and associated statistics is provided in Supplementary Data 9.

### A subset of expressed receptors is implicated in metabolic disorders

**Fig. 6:**
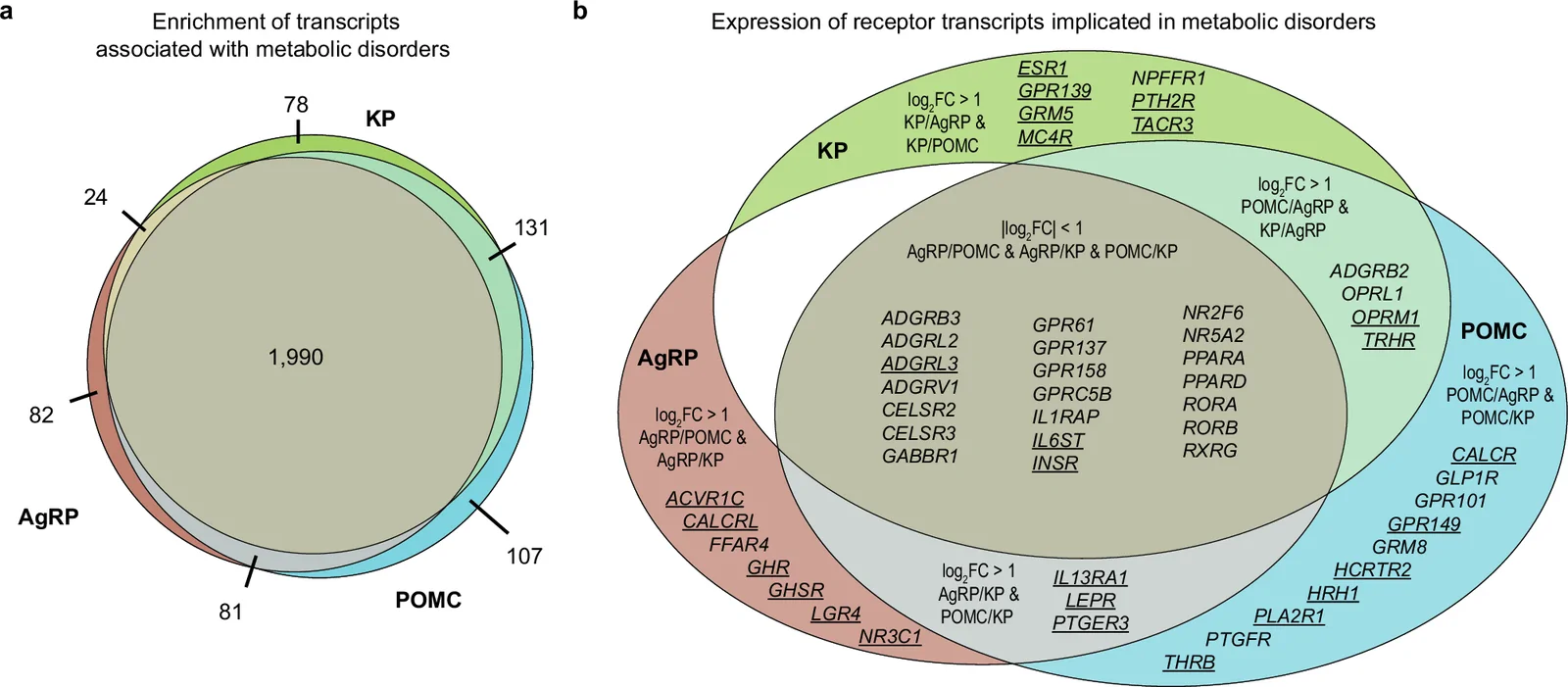
Expression of transcripts associated with metabolic disorders in AgRP, POMC, and KP neurons. **a** Distribution of 2493 metabolic phenotype-associated transcripts (GWAS Catalog database; Supplementary Data 11) across AgRP, POMC, and KP neurons. **b** Venn Diagram illustrating 52 receptor mRNAs (KEGG Brite database; Supplementary Data 12) linked to metabolic disorders that exhibit cell type-specific or shared expression. Underlined gene symbols denote statistically significant differential expression (*P*.adj. <0.05, DESeq2, two-sided Wald test with Benjamini–Hochberg FDR correction) between any two cell types. Gene sets showing at least twofold enrichment (log_2_ fold change ≥1) were categorized as either cell type-specific or shared between two cell types relative to the third. (Genes in the largest intersection showed no preferential enrichment in any single neuronal phenotype).

To analyze transcripts associated with metabolic disorders, we selected 14 metabolism-related phenotypes (Table 1) from the GWAS Catalog^13^. From the corresponding gene sets listed in Supplementary Data 10, we identified 2493 transcripts in the AgRP-, POMC-, and/or KP neuron transcriptomes, applying cutoffs of mean TPM ≥ 10 and reads >5 in each sample of the respective cell type (Fig. 6a and Supplementary Data 11). Of these, 52 receptor genes were represented in the KEGG BRITE database and showed either cell type-specific enrichment or shared expression across the three neuronal phenotypes (Fig. 6b and Supplementary Data 12). To identify potential therapeutic targets for metabolic disorders, we further examined the receptor profiles.

**Table 1:** Transcripts associated with metabolic disorders in AgRP, POMC, or KP neurons

Mapped traits (GWAS Catalog)	Significant SNPs (*P *< 5 × 10^−8^)	Affected genes (mapped to SNPs)	Expression in AgRP, POMC or KP cells
Abdominal adipose tissue measurement	44	18	8
BMI-adjusted hip circumference	4541	961	513
BMI-adjusted waist circumference	5997	1068	562
Body Fat Percentage	1391	478	280
Body mass index	18,537	2397	1445
Body weight (without child traits)	5456	684	412
Eating disorder	233	149	98
Fat pad mass	982	432	266
Hip circumference	257	98	54
Metabolic syndrome	2415	1027	629
Overnutrition	632	164	73
Visceral adipose tissue quantity	302	164	105
Waist circumference	264	97	54
Waist-Hip ratio	8750	1133	611

Summary of gene-assigned single nucleotide polymorphisms (SNPs), associated genes, and the number of corresponding transcripts we identified in AgRP-, POMC-, and/or KP neuron transcriptomes for each metabolic trait (GWAS Catalog database; Supplementary Data 10 and 11). Cutoffs: mean TPM ≥ 10 and reads ≥5 in transcriptomes of any neuronal phenotype.

### Receptor profiles reveal humoral and neuronal regulatory mechanisms

**Fig. 7:**
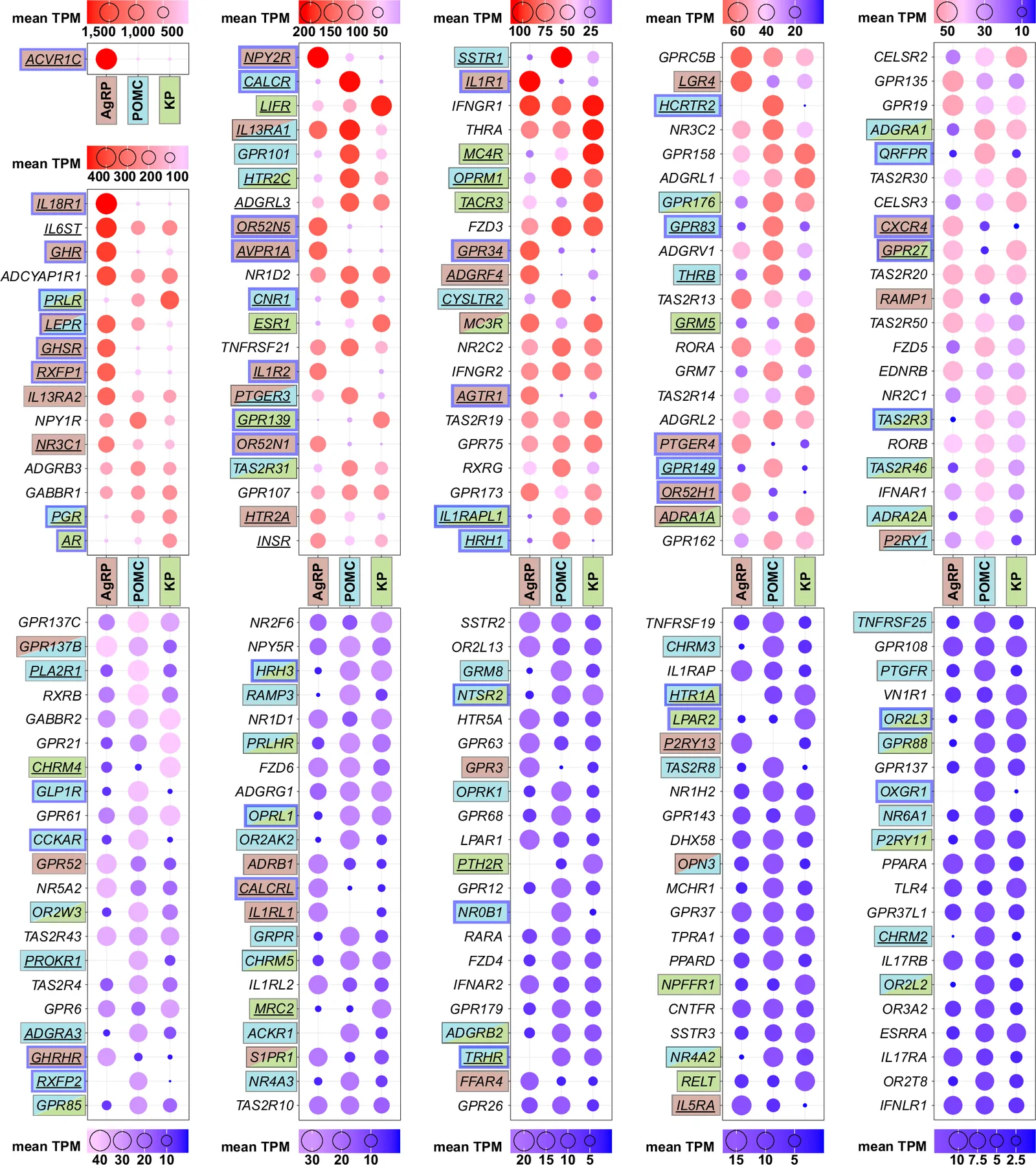
Receptor profiles of human AgRP, POMC, and KP neurons. Dot plots of 205 receptor transcripts (cutoffs: mean TPM ≥ 10 and reads ≥5 in each sample of the same cell type). Dot size and color are scaled according to transcript abundance. Underlined symbols denote statistically significant differences between any two cell types (*P*.adj. <0.05, DESeq2, two-sided Wald test with Benjamini–Hochberg FDR correction), whereas single or double text shading indicates at least twofold enrichment (log_2_ fold change ≥1). Genes with greater than fourfold enrichment (log_2_ fold change >2) are outlined in blue. See also Supplementary Data 12 for TPM values and statistical results.

A total of 205 receptor transcripts detectable in human AgRP-, POMC-, and/or KP-IR neurons (cutoffs: mean TPM ≥ 10 and reads ≥5 in each sample of the same cell type) are illustrated in Fig. 7 and listed in Supplementary Data 12. These datasets identify candidate receptors that could be targeted to modulate hypothalamic metabolic and/or reproductive pathways. A total of 112 receptors showed at least twofold enrichment, and 62 were significantly differentially expressed in pairwise comparisons between neuronal phenotypes. *ACVR1C* encoding an activin receptor was the most abundantly expressed receptor, occurring exclusively in AgRP neurons. This transcriptome also showed selective enrichment for receptors of proinflammatory cytokines (*IL18R1* and *IL1R1*), circulating hormones (*GHR*, *GHSR*, and *NR3C1*), and various neuropeptides related to energy homeostasis (*RXFP1*, *NPY2R*, *AVPR1A*, *MC3R*, *AGTR1*, and *CALCRL*), as well as three olfactory receptors: *OR52N5*, *OR52N1*, and *OR52H1*. The POMC neuron transcriptomes exhibited elevated expression of receptors involved in metabolic hormone and neuropeptide signaling, including *NPY1R*, *CALCR*, *SSTR1*, *HCRTR2*, *QRFPR*, *GLP1R*, *CCKAR, PROKR1*, and *GRPR*, as well as the type 1 endocannabinoid- (*CNR1*), the type 2 serotonin- (*HTR2C*), and the type 1 histamine (*HRH1*) receptors. The leptin receptor (*LEPR*) was co-enriched in AgRP and POMC cells. POMC and KP neuron transcriptomes both expressed reproductive hormone receptors (*PRLR*, *PGR*, *AR*, and *ESR1*), with higher levels in the latter, whereas opioid receptors (*OPRM1*, *OPRL1*, and *OPRK1*) were expressed more prominently in POMC than in KP neurons. *IL6ST, ADCYAP1R1*, and the insulin receptor *(INSR)* were expressed highly in all three cell phenotypes (Fig. 7 and Supplementary Data 12).

Among the orphan GPCRs, GPR34, GPR27 (SREB1), and GPR3 were enriched in AgRP neurons, GPR101, GPR176, GPR83, GPR149, and GPR85 (SREB2) were expressed in POMC neurons, and GPR107, GPR75, GPRC5B, GPR158, GPR162, GPR135, GPR19, GPR137C, GPR137B, GPR61, and GPR6 showed shared expression across both neuronal phenotypes (Fig. 7 and Supplementary Data 12).

### Neuropeptide and receptor profiles differ markedly in humans and mice

**Fig. 8:**
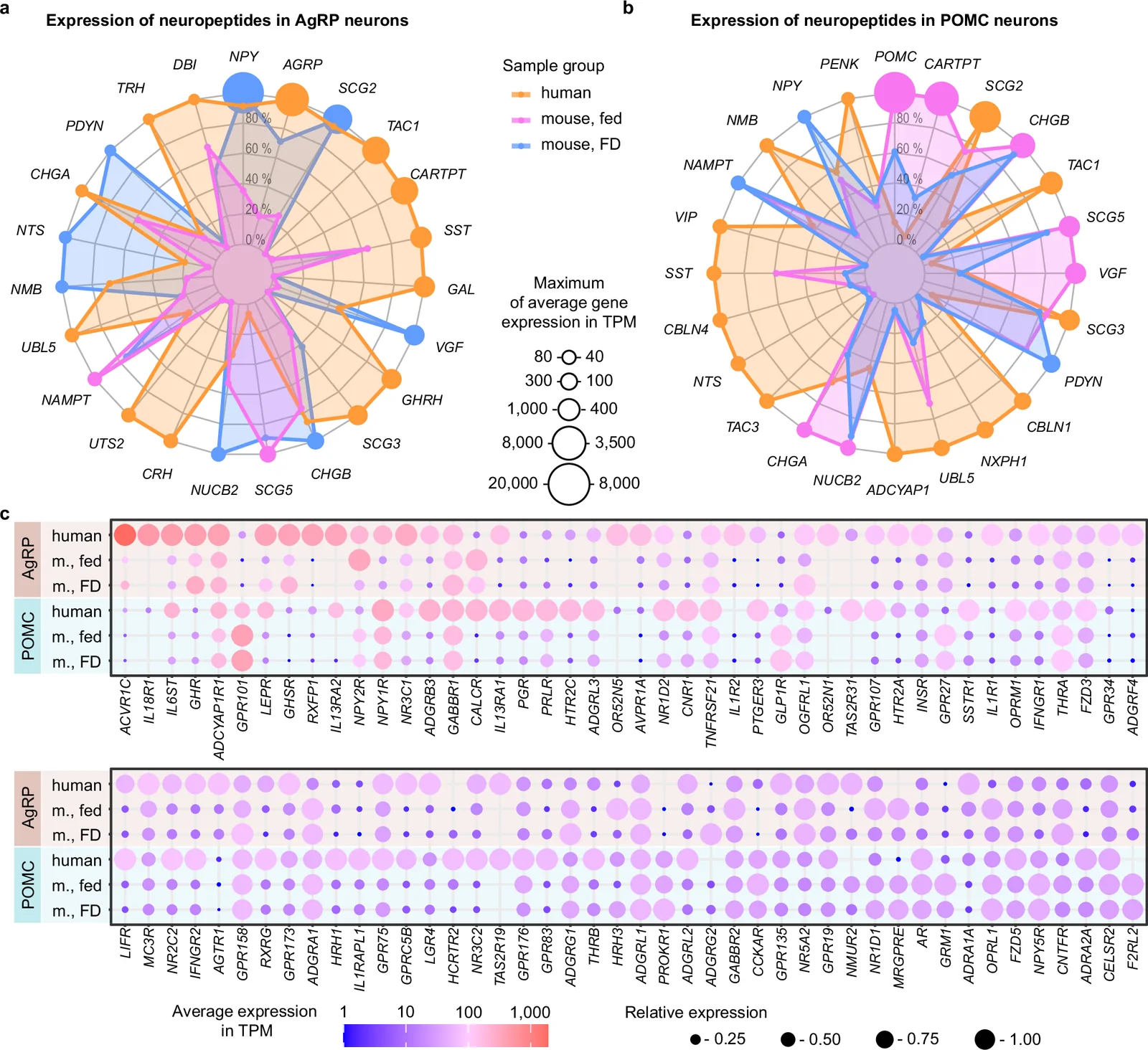
Species-specific expression patterns of neuropeptides and receptors in human and mouse AgRP and POMC neuron transcriptomes. **a**, **b** Radar charts showing neuropeptides expressed at mean TPM ≥ 40 in AgRP (**a**) and POMC (**b**) neurons in humans, and in mice from 24-h food-deprived (FD) and from ad libitum-fed states. **c** Bubble plots illustrating species-dependent expression of 88 receptors, compiled from the most abundant 40 receptors of each transcriptome type. For expression values in TPM, see Supplementary Data 13-15.

Earlier bulk sequencing studies from other laboratories defined the gene expression profiles of murine AgRP and POMC neurons in detail, providing a framework for assessing similarities and differences between mice and humans. To enable cross-species comparisons, we re-analyzed publicly available raw RNA-seq datasets from AgRP and POMC neurons reported for both *ad libitum*-fed and 24-h food-deprived (FD) male mice^14^ and converted expression values to TPM. Supplementary Data 13 presents data for all recognized orthologs detected in human and murine hypothalamic AgRP and POMC neuron transcriptomes. Analysis of neuropeptides with mean expression ≥40 TPM in human or mouse AgRP or POMC neuron transcriptomes, including mouse fed and food-deprived states, revealed pronounced species differences. Accordingly, *TAC1*, *CARTPT*, *GAL*, *GHRH*, *CRH*, *UTS2*, *UBL5*, and *TRH* were uniquely expressed in human AgRP neurons, whereas *Scg5* and *Nampt*, which were present in murine AgRP transcriptomes, were undetectable in their human counterparts. *Nts* and *Pdyn* expression were detectable only in food-deprived mice (Fig. 8a and Supplementary Data 14). Regarding POMC neurons, *TAC1*, *CBLN1*, *NXPH1*, *ADCYAP1*, *TAC3*, *NTS*, *CBLN4*, *VIP*, *NMB*, and *PENK* were enriched in humans, whereas *Cartpt, Chgb, Scg5*, and *Pdyn* were enriched in mice (Fig. 8b and Supplementary Data 14).

For further species and cell-type comparisons, we also analyzed the 40 most abundant receptors from each of the six transcriptomes, yielding 88 receptors in total (Fig. 8c and Supplementary Data 15). Among the top receptors of human AgRP neurons, *ACVR1C*, *IL18R1*, *IL6ST*, *RXFP1*, *IL13RA1*, and *HTR2A* showed only low expression in mouse AgRP neurons, whereas *Calcr* was markedly more abundant in this cell type of mice. Human POMC neurons likewise expressed substantially higher levels of *IL6ST, LEPR, IL13RA2, ADGRB3*, *CALCR*, and *HTR2C* than their murine counterparts. In contrast, *Gpr101* and *Glp1r* expression levels in POMC neurons were higher in mice than in humans. The striking species differences underscore the importance of directly examining metabolically relevant neuronal phenotypes in the human.

### Spatial context shapes the transcriptome of human POMC neurons

**Fig. 9:**
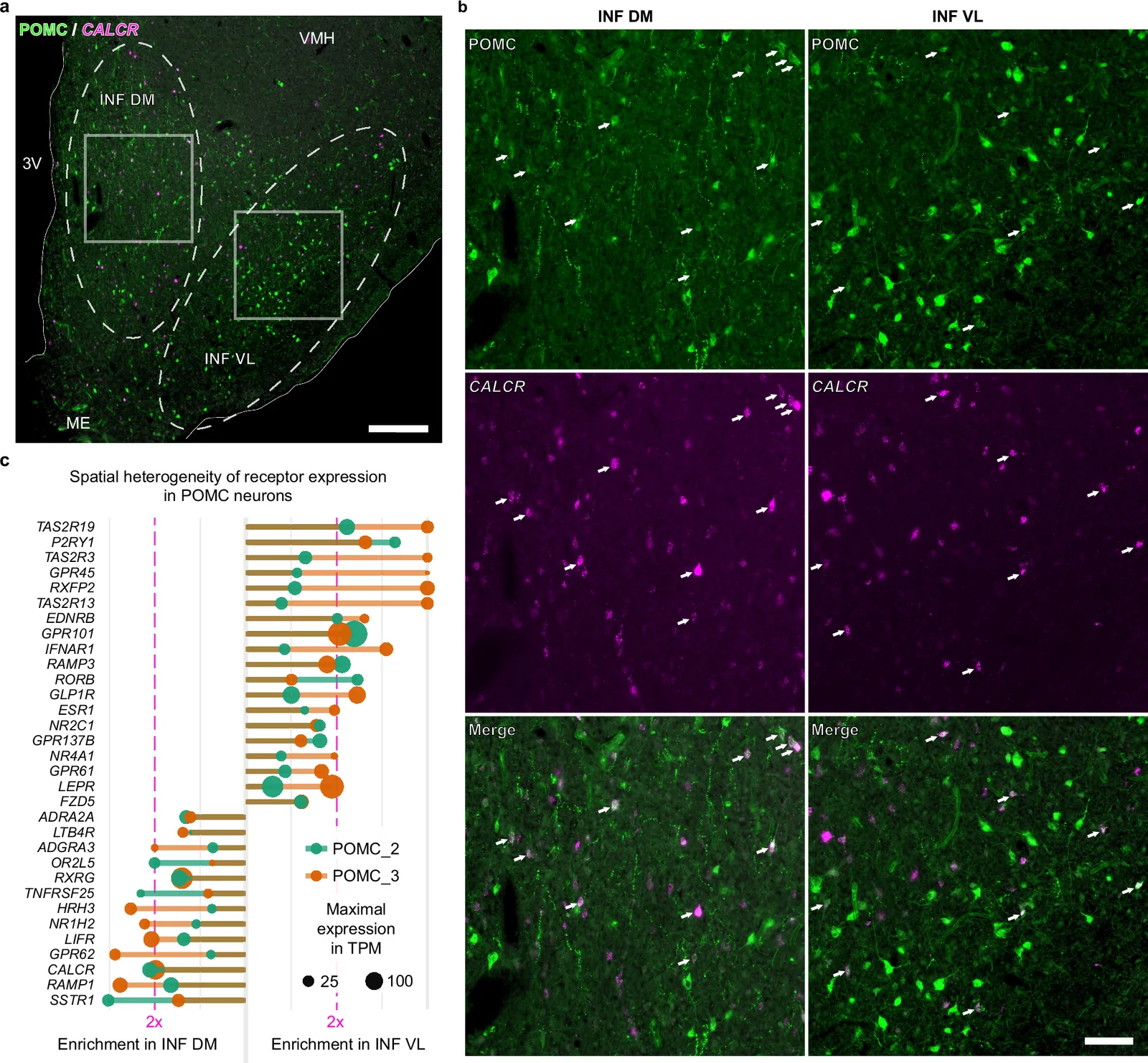
Spatial heterogeneity of the human POMC neuron transcriptome. According to plate 27 of Mai et al.^15^, POMC-IR neurons in the infundibular nucleus (INF) are organized into dorsomedial (DM) and ventrolateral (VL) anatomical subpopulations that differ in their gene-expression profiles. **a**, **b** RNAscope in situ hybridization detection of *CALCR* mRNA (magenta) in 21.14% and 11.76% of POMC neurons (green) in the DM and VL subregions of the INF, respectively. Arrows in (**b**), corresponding to the boxed areas in (**a**), indicate double-labeled neurons. **c** Preferential receptor gene expression in DM and VL POMC-IR cell groups, as revealed by IHC/LCM-Seq. Dot size indicates expression level in TPM in samples from two human subjects (POMC_2 and POMC_3; Samples #6 and #9, respectively, in Supplementary Data 3). Receptors were included if they showed ≥5 reads and ≥10 TPM in either the DM or VL group in both individuals, an absolute DM–VL log_2_ FC > 0.2 in each subject, and a mean absolute log_2_ FC > 0.5 across subjects. 3V 3rd ventricle, INF DM dorsomedial part of the infundibular nucleus, INF VL ventrolateral part of the infundibular nucleus, ME median eminence, VMH ventromedial hypothalamic nucleus. Scale bars: 400 µm in (**a**) and 100 µm in (**b**). For full transcriptomes, see Supplementary Data 16.

IHC/LCM-Seq relying on bulk sequencing provides composite transcriptomic information from potential neuronal subpopulations within a given neuronal phenotype. The recent HYPOMAP study^3^ distinguished three POMC neuron subtypes, each defined by a distinct set of marker genes. Among these, *CALCR* emerged as a hallmark of cluster C4-374, the POMC-CALCR cluster, which is distributed preferentially near the third ventricle. To address spatial heterogeneity among POMC neurons, we combined immunofluorescent visualization of POMC perikarya with RNAscope in situ hybridization for *CALCR* mRNA in three sections corresponding to plate 27 of the human brain atlas^15^ (Sample #6 in Supplementary Data 3). The in situ hybridization signal for the receptor was detected in 54 of 361 POMC-IR neurons (14.96%; Fig. 9a, b). POMC neurons formed two topographically distinct cell masses in this coronal plane (Fig. 9a, b). *CALCR* co-expression was observed in 21.14% of the dorsomedial (DM) POMC neuron subpopulation (26/123 neurons) and 11.76% of the ventrolateral (VL) POMC neuron subpopulation (28/238 neurons). The modest enrichment of dual-labeled neurons closer to the third ventricle corroborated results of the HYPOMAP study^3^. To capitalize on the high spatial resolution of the IHC/LCM-Seq method, we further analyzed the anatomically distinct DM and VL subpopulations of POMC neurons (300–500 neurons per region per subject) collected by LCM from immunostained sections of 28- and 57-year-old individuals (samples #6 and #9; Supplementary Data 3). Our transcriptomic analysis (Supplementary Data 16**;** PRJNA1453244) identified 1052 transcripts that were enriched at least twofold in either the DM or VL POMC neuron transcriptomes in both brain samples, including 339 DM-enriched and 713 VL-enriched transcripts (cutoffs: mean TPM ≥ 10; reads ≥5 in each subject). Figure 9c illustrates selected receptor genes with pronounced differential enrichment between the DM and VL POMC neuron subpopulations. The higher abundance of *CALCR* and *SSTR1* in the DM subgroup was consistent with the spatial distribution of C4-374 in the HYPOMAP study. In contrast, *LEPR*, *RXFP2*, *GPR101*, and *GLP1R*, which were identified as typical transcripts of the main POMC neuron cluster C4-373, were enriched more in the VL subpopulation^3^. *RAMP1* was enriched in the DM subgroup and *RAMP3* in the VL subgroup, suggesting that coupling with *CALCR* may favor AMY_1_ and AMY_3_ amylin receptor formation, respectively, in these two neuronal subsets.

## Discussion

Our study provided high-resolution insight into the molecular architecture of orexigenic AgRP and anorexigenic POMC neurons regulating energy homeostasis. The inclusion of KP neurons in our analysis as a third cell type offered a valuable comparative perspective; while localized similarly in the INF, KP neurons play a primary role in regulating the reproductive axis^7^. With 14,000–16,000 transcripts detected per neuronal phenotype, 1210 of which were differentially expressed, these three datasets constitute a comprehensive resource for dissecting the transcriptional programs underlying mediobasal hypothalamic control of energy balance and reproduction.

The methodological framework underlying the transcriptomic studies of neuronal cell types from *postmortem* human brain tissue was adapted from the previously established LCM-Seq method, which was originally developed for spatial transcriptomic profiling of fluorescently labeled neurons in transgenic mice^16^. To overcome the need for genetic tagging, we subsequently developed the more versatile IHC/LCM-Seq method, which replaced transgenic labeling of murine cell types with RNA-preserving IHC. This change enabled comprehensive molecular profiling of neuronal phenotypes across species^6^. Here, we adapted this IHC/LCM-Seq technique to *postmortem* human brain tissue, marking a major leap forward in translational applications. This transition introduced some unique challenges inherent to autopsy-derived material, necessitating the implementation of rigorous quality control measures throughout the workflow. We first confirmed that tissue samples collected within 24 h *postmortem* and immersion-fixed in cold 4% FA for 24–72 h preserved the integrity of RNA sufficiently. Further, we found that variations within this fixation window had minimal—if any—impact on RNA quality, offering practical flexibility for tissue processing. Importantly, the number of detectable transcripts remained largely unaffected by the initial RIN values, which ranged from 3.1 to 6.7 in RNA-seq studies of AgRP-, POMC-, and KP-IR neurons. We attributed this robustness to the use of random primers in the TruSeq library preparation protocol, which was reported to accommodate even highly fragmented RNA^17^. In subsequent tissue handling (post-fixation, 20% sucrose infiltration, sectioning at 20 µm, and storage at –20 °C in cryoprotectant solution containing 0.05% ATA), we adhered to the protocols we previously optimized for perfusion-fixed mouse and rat brains^6^. When working with immersion-fixed human brains, successful immunolabeling may require an epitope retrieval step (30 min at 80 °C in 0.1 M citrate buffer, pH 6.0). We found that this pretreatment severely compromised RNA quality; however, the addition of 0.05% ATA or 10% PVSA to the retrieval buffer effectively mitigated RNA degradation. Subsequent immunostaining in the presence of either 0.05% ATA or 2% PVSA efficiently preserved RNA integrity at both 4 °C and RT. However, ATA increased nonspecific background staining and compromised the specific signal, making PVSA the preferred RNase inhibitor for routine IHC/LCM-Seq applications. Overall, our methodological results confirmed that IHC/LCM-Seq is a robust, highly sensitive, and species-independent technique. Adapting this method to *postmortem* human brains in this study will have substantial translational and clinical relevance.

We propose that IHC/LCM-Seq and single-cell approaches are complementary methodologies, each offering unique advantages depending on the biological question. As discussed previously^6^, IHC/LCM-Seq offers several advantages over single-cell approaches, which typically require dissociation or nuclear isolation from brain tissue. (i) LCM preserves the spatial context of neurons, maintaining their native anatomical architecture and microenvironment. (ii) Tissue fixation prior to RNA extraction stabilizes the transcriptome and reduces artifacts introduced during the isolation process. (iii) IHC/LCM-Seq defines neuronal phenotypes based on immunohistochemical detection of neurotransmitter markers. In contrast, single-cell approaches typically rely on transcriptomic clustering, which may overlook the fact that RNA expression does not necessarily reflect protein abundance. (iv) Neuronal pools used in IHC/LCM-Seq can retain subject-level transcriptomes as independent biological replicates. In contrast, single-cell approaches commonly define cell-type clusters from mixed samples, often with disproportionate contributions from multiple donors. Most importantly, (v) IHC/LCM-Seq provides markedly enhanced sensitivity for detecting low-abundance transcripts. Rare transcription factors, lncRNAs, and several receptor mRNAs detected in our datasets, including *CCKAR*, *IL1RL1*, *NPY2R*, *OR52N5*, and *PROKR1*, remain inaccessible or poorly accessible to single-cell techniques^3^.

We note that achieving this level of transcriptome coverage involves a trade-off in cellular resolution. Unlike single-cell methods, bulk RNA sequencing cannot resolve cellular heterogeneity or distinguish subpopulations within a given neurotransmitter phenotype. Accordingly, heterogeneity among POMC-IR neurons necessarily remained unexplored in our bulk datasets. The recent HYPOMAP study, which combined large-scale single-nucleus transcriptomics with spatial transcriptomics to generate an integrated spatiocellular atlas of the human hypothalamus, identified three transcriptionally distinct human POMC neuron clusters. *CALCR* emerged as a notable marker gene for cluster “C4-374”. These neurons are preferentially located close to the third ventricle and represent the second-largest POMC cluster, accounting for nearly 5% of human POMC neurons based on “mean regional abundance score”^3^. Taking into account these data, we supplemented our studies with spatial methods capable of addressing POMC neuronal heterogeneity by combining RNAscope in situ hybridization for *CALCR* with immunohistochemical detection of POMC. Microscopic analysis established that *CALCR*-positive POMC-IR neurons represent a minority population (~ 15%) that intermingles with *CALCR*-negative neurons throughout the INF. Their incidence is approximately twice as high in the DM subdivision as in the VL subdivision of the nucleus, supporting a similar pattern from the HYPOMAP study. In a second series of IHC/LCM-Seq studies, we took advantage of the method’s spatial resolution to determine whether POMC neurons in the DM and VL subdivisions of the INF display distinct gene-expression landscapes. This analysis identified hundreds of transcripts that were consistently enriched in POMC neurons from either the DM or VL subdivision of the INF, corroborating the idea that spatial context shapes the gene-expression profile of the same neuronal phenotype.

Transcription factors likely play important roles in defining the individual molecular features of the three neuronal phenotypes. Their detailed analysis revealed both shared and cell-type-specific characteristics. Although AgRP, POMC, and KP neurons fulfill distinct functions, their TFs overlapped substantially, potentially reflecting common developmental origins^18^ and/or microenvironmental influences. The presence of sex steroid receptors (*PGR*, *AR*, *ESR1*) in both the POMC and the KP neuron transcriptomes provides a potential mechanistic link between gonadal hormone signaling and hypothalamic regulation of metabolic functions^19,20^. Beyond similarities, 50 of the 902 detected TFs were differentially expressed across the three neuronal phenotypes.

Among the co-expressed neuropeptides, *CARTPT*, encoding the neuropeptide CART, was detected in AgRP-IR neurons but not in POMC cells. This pattern highlights a notable species difference from rodents that was not emphasized in the HYPOMAP study^3^ but was reported previously^11^ and confirmed in the present study using immunofluorescence. We also detected high levels of *CARTPT* mRNA in KP neurons, consistent with our earlier immunohistochemical observations^21^. It is worth noting that AgRP neurons were reported to form only a single cluster in the HYPOMAP study^3^. In contrast, our immunofluorescence colocalization experiments established that different neuropeptides (Neuropeptide Y, Substance P, CART, somatostatin, and galanin) are co-expressed in AgRP-IR neurons to highly variable degrees, providing clear evidence of considerable neurochemical heterogeneity within this cell type. These peptides may play thus far underappreciated roles in human orexigenic circuitry.

In this study, we paid special attention to disease-associated genes expressed in the orexigenic and/or anorexigenic neuronal phenotypes. Somewhat unexpectedly, we found that, among the 2493 transcripts associated with metabolic traits in the GWAS Catalog^13^, the majority (1990) exhibited very similar expression across all cell types. Only 503 showed twofold or greater enrichment in any specific cell type, suggesting that many GWAS-linked genes exert their effects across multiple hypothalamic pathways.

Receptors may represent potential targets for metabolic therapies. Therefore, a key objective of our study was to characterize the receptor expression landscape of these neuronal phenotypes. We found that many receptor genes, including those listed in the GWAS Catalog^13^, were expressed across multiple cell types. Among all receptors, *ACVR1C* (ALK7) was present at the highest level in AgRP neurons. Genetic loss of this receptor or its natural ligand activin B in mice reduces the number of Npy-expressing cells, decreases *Npy* and *AgRP* mRNA levels, and diminishes NPY/AgRP projections to the MPOA, thereby supporting a trophic role for Alk7 in maintaining orexigenic circuitry^22^. AgRP-IR neurons highly expressed metabolic hormone receptors, including *GHSR* (for ghrelin), *INSR* (for insulin), and several cytokine receptors (*IL18R1*^23^, *IL1R1*^24^) acting through the MyD88-dependent NF-κB and MAPK signaling cascade, and other receptors using JAK-STAT signaling (*GHR*^25^ and *LEPR*^26^*, IL6ST* and *IL13RA1*^27^). These receptors indicate that AgRP neurons serve as a key cell type of immune-metabolic integration^28^. *ADCYAP1R1*, which encodes the PACAP receptor, was highly expressed across cell types. PACAP may exert a dual anorexigenic effect, analogous to leptin, by inhibiting AgRP neurons and activating POMC neurons, thereby promoting melanocortin signaling to downstream targets such as the paraventricular hypothalamus^29^. The relaxin receptor *RXFP1* was also abundant in AgRP cells, while POMC neurons expressed *RXFP2*. Studies in rats found reduced food intake following peripheral or central administration of relaxin^30^. The occurrence of the V1a vasopressin receptor *AVPR1A* in AgRP neurons may link hydration or stress state to feeding drive^31^. The angiotensin II receptor *AGTR1* we detected in AgRP neurons may play an important role in resting metabolic rate adaptation, a mechanism that likely contributes to failed weight management during obesity^32^. The interesting presence of three olfactory receptors in AgRP neurons suggests potential responsiveness to yet-unidentified circulating metabolites or microbiota-derived compounds. POMC neurons abundantly expressed *PRLR*, suggesting sensitivity to prolactin signaling through the JAK-STAT pathway. The concomitant expression of *LEPR*, *IL6ST*, and *IL13RA1* further indicates that POMC neurons may integrate multiple cytokine- and hormone-related signals that influence JAK-STAT-associated pathways, potentially contributing to the coupling of reproductive status with metabolic regulation. Calcitonin, acting via the calcitonin receptor (*CALCR*), may synergize with leptin signaling to suppress food intake and promote satiety. The presence of *RAMP* transcripts, in addition to *CALCR*, suggests that POMC neurons may be capable of forming AMY1 and AMY3 amylin receptor complexes, raising the possibility that pancreas-derived amylin, co-secreted with insulin by β-cells, acts on these neurons to promote satiety and reduce food intake^33^. AgRP neurons, but not POMC neurons, expressed both *CALCRL* and *RAMP1*, the core components required for the canonical CGRP receptor complex. The physiological sources and functional relevance of calcitonin-like ligands acting on POMC neurons and CGRP acting on AgRP neurons remain to be clarified. POMC neurons expressed high levels of the serotonin receptor *HTR2C*, which may contribute to the anorexigenic actions of serotonin and the selective 5-HT2C agonist anti-obesity drug lorcaserin^34^. This cell type also showed abundant expression of *CNR1*, encoding the CB1 cannabinoid receptor. In mice, endocannabinoid responsiveness of POMC neurons^35^ has been proposed to enhance appetite, at least in part by modulating glutamatergic signaling to the paraventricular nucleus^36^.

Our study identified similarities as well as robust species differences between laboratory mice and humans by re-analyzing publicly available raw RNA-seq datasets from murine AgRP and POMC neurons^14^. Such cross-species bulk transcriptomic comparisons are limited by orthologue availability and differences in phenotype definition, with IHC-based identification in humans versus transgenic labeling in mice. Clinically relevant similarities between the two species are exemplified by the predominant expression of *GLP1R* in POMC neurons, which may play a key role in the central mechanisms of appetite suppression by semaglutide, a GLP-1 receptor agonist used for the treatment of obesity and diabetes^37,38^. Human AgRP neurons also contained *GLP1R* at low levels, in accordance with recent observations in mice, where orexigenic neurons express GLP-1R protein and receive GLP-1R-IR afferent input^39^. Beyond common features, notable differences between the two species highlight important limitations of the rodent models. Likewise, orexin A stimulates endocannabinoid signaling in mouse POMC cells via OX-1R^40^, whereas in humans, OX-2R, encoded by *HCRTR2*, may mediate analogous effects. The selective expression of *OR52N5*, *OR52N1*, and *OR52H1* in human AgRP neurons highlights species-specific features that may be relevant for developing future pharmacological strategies for weight management.

In conclusion, we establish IHC/LCM-Seq as a robust approach for transcriptomic profiling of immunohistochemically defined neuronal phenotypes in immersion-fixed human *postmortem* brain tissue. Applied to AgRP-, POMC-, and KP-IR neurons of the INF, this strategy delineates molecular features of human hypothalamic circuits involved in metabolic and reproductive regulation, identifies species-specific differences from widely used rodent models, and supports the use of *postmortem* human brain tissue for defining clinically relevant neuroendocrine targets.

## Methods

### Human subjects

Histological samples from adult human brains (*N* = 13) were obtained during autopsies conducted at the Department of Pathology and Experimental Cancer Research, Semmelweis University, Budapest, Hungary. The specimens were derived from one 53-year-old female and from male individuals aged 28, 33, 35, 38, 45, 46, 49, 50, 52, 53, 56, and 57 years, all with no documented neurological disorders (Supplementary Data 3). Donors were of European ancestry/ethnicity (Hungarian). Socioeconomic status was not collected due to the de-identified nature of the samples and the absence of these fields in the source records. We note that the lack of socioeconomic status data and the male-only analytic dataset of European-ancestry adults may constrain the generalizability of the findings to other populations. Ethical approval was granted by the Regional and Institutional Committee of Science and Research Ethics of Semmelweis University (SE-TUKEB 251/2016), in compliance with the Declaration of Helsinki and relevant Hungarian legislation (Act CLIV of 1997 and Decree 18/1998 [XII.27.] of the Ministry of Health), which does not mandate prior written consent from the deceased.

### Sample selection for IHC/LCM-Seq and RNAscope in situ hybridization studies

*Postmortem* time was kept below 24 h. Due to variability in staining quality, not all hypothalamic specimens could be included in all downstream analyses, resulting in AgRP, POMC, and KP neuron transcriptomes from 3, 2, and 3 subjects, respectively. For spatial transcriptomic analyses comparing POMC-IR neurons in the DM and VL subdivisions of the INF, specimens were obtained from two human subjects (#6 and #9; Supplementary Data 3).

### IHC/LCM-Seq and RNAscope in situ hybridization experiments

For all experiments, reagents were of molecular biology grade. MQ water and PBS were pretreated overnight with diethylpyrocarbonate (DEPC; Thermo Scientific; 1 mL/L) and subsequently autoclaved. All other solutions were prepared using DEPC-treated and autoclaved MQ water. Reagents and buffers were maintained under RNase-free, sterile conditions with or without the additional use of the RNase inhibitors. Work surfaces were cleaned with RNaseZAP (Merck KGaA, Darmstadt, Germany).

### Optimization of IHC/LCM-Seq for human tissue

#### Immersion fixation and section preparation

To optimize the IHC/LCM-Seq protocol for brain samples gained from autopsies, test experiments were performed using 4-mm-thick tissue blocks (*n* = 9) dissected 20 h *postmortem* from the frontal cortex of a 53-year-old female subject. Samples were immersion-fixed for 24, 48, or 72 h (*n* = 3 per fixation time) in freshly prepared ice-cold 4% formaldehyde (FA) in 0.1 M phosphate-buffered saline (PBS; pH 7.4). Following fixation, tissues were infiltrated with ice-cold sterile 20% sucrose in PBS for 24 h, snap-frozen on powdered dry ice, and stored at -80 °C until sectioning. The fixative and sucrose solutions were supplemented with 0.05% aluminon (triammonium salt of aurintricarboxylic acid, ATA; Thermo Fisher Scientific, Waltham, MA, USA) to enhance RNA preservation^6,8^. Prior to sectioning, the white matter was trimmed from each tissue block. Free-floating 20-µm-thick sections were prepared using a Leica freezing microtome (Leica Biosystems, Wetzlar, Germany) (Fig. 1a).

#### Assessing the impact of immersion fixation

RNA was extracted from individual cortical sections fixed for 24, 48, or 72 h using the PureLink FFPE RNA Isolation Kit (Thermo Fisher Scientific). For each fixation time (*n* = 3 experimental replicates per fixation time), RNA yield—normalized to the section area (mm^2^)—and RNA Integrity Number (RIN) were determined using the Agilent 2100 Bioanalyzer system with Eukaryotic Total RNA Pico Chips and the 2100 Expert software (Agilent, Santa Clara, CA, USA). Results were presented as mean ± SEM from triplicate measurements (Supplementary Data 1).

#### RNA preservation during antigen retrieval

Most cortical sections were stored for one month at −20 °C in a modified cryoprotectant solution (30% ethylene glycol, 25% glycerol, 0.05 M phosphate buffer; pH 7.4) supplemented with 0.05% ATA. We have previously demonstrated that this storage method preserves RNA integrity for 3 years or longer^6^. Optimization of section pretreatments and the immunohistochemical protocol used the three tissue blocks that were immersion-fixed for 72 h. Sections retrieved from the antifreeze medium were rinsed in sterile PBS and subjected to a 30-min heat-induced epitope retrieval step in 0.1 M citrate buffer (pH 6.0, 80 °C), either without additives or in the presence of the RNase inhibitors ATA (0.05%) or polyvinyl sulfonic acid (PVSA; Merck)^41^ used at 1%, 3%, or 10%. Following brief PBS washes, total RNA was extracted, and RNA integrity was assessed using the Agilent 2100 Bioanalyzer system. Results were presented as mean ± SEM (Supplementary Data 2) of three measurements on independent samples. Statistical differences between treatment groups—with and without antigen retrieval—were assessed by one-way ANOVA followed by Tukey’s post hoc tests.

#### Optimization of the RNA-preserving immunohistochemistry

Antigen retrieval was performed in the presence of 10% polyvinyl sulfonic acid (PVSA), which efficiently protected RNA during method optimization. Subsequently, sections were processed for immunohistochemical detection of cortical parvalbumin-expressing neurons at either RT or 4 °C, with or without the use of RNase inhibitors. To inhibit RNases, all antibody and buffer solutions (except the peroxidase developer) contained either 0.05% aluminon (ATA) or 2% PVSA. Sections were pretreated with 1% H_2_O_2_ and 0.5% Triton X-100 in PBS for 20 min, followed by incubation with a commercially available affinity-purified goat polyclonal antibody against human parvalbumin (#EB06776, P1; Everest Biotech; 1:30,000) for either 40 h at 4 °C or overnight at RT. The primary antibody was reacted with Biotin-SP-AffiniPure anti-goat IgG secondary antibody (Jackson ImmunoResearch; 1:500; overnight at 4 °C or 2 h at RT), followed by the ABC Elite reagent (Vector Laboratories, Burlingame, CA, USA; 1:1000; 1 h at RT) in PBS. The peroxidase reaction was visualized at RT using a developer containing 10 mg diaminobenzidine, 30 mg nickel-ammonium sulfate, and 0.003% H_2_O_2_ in 20 ml of 0.05 M Tris-HCl buffer (pH 7.6). RNA was extracted from pre-IHC sections (*n* = 3) and from post-IHC sections (*n* = 3 per treatment group, experimental replicates), and RNA integrity was assessed using the Agilent 2100 Bioanalyzer System (Supplementary Data 2). Differences in RIN values among the post-IHC groups were analyzed using one-way ANOVA followed by Tukey’s post hoc tests. For reagents, see Supplementary Data 8.

For qualitative analysis of the labeling, the immunostained sections were mounted onto microscope slides from Elvanol, air-dried for 30 min, then sequentially dehydrated in 70%, 95%, and 100% ethanol (5 min each), followed by clearing in xylene (2 × 5 min). Coverslipping was performed using DPX mounting medium (Merck) for light microscopic analysis and imaging. Digital images were adjusted for brightness and contrast using Adobe Photoshop (v12.0; Adobe Systems), and final composite figures were assembled in Adobe Illustrator (v29.7.1; Adobe Systems).

### IHC/LCM-Seq studies of hypothalamic neurons

#### Immersion fixation of hypothalamic samples

Four-mm-thick hypothalamic slices from 8 male individuals (Samples #2–9, Supplementary Data 3) were immersion-fixed in ice-cold 4% FA in 0.1 M PBS (pH 7.4) for 72 h, followed by infiltration in 20% sucrose (24 h). Both solutions were supplemented with 0.05% aluminon (ATA). Free-floating 20-µm-thick coronal sections were prepared from the mediobasal hypothalamus (atlas plates 24–27)^15^ using a freezing microtome and stored in modified cryoprotectant solution containing 0.05% ATA, as described for cortical samples.

#### RNA quality testing

Total RNA was extracted from a hypothalamic section per case using the PureLink FFPE RNA Isolation Kit (Thermo Scientific). Its integrity was assessed with the Agilent 2100 Bioanalyzer system. Samples used in subsequent IHC/LCM-Seq experiments exhibited RNA Integrity Number (RIN) values ranging from 3.1 to 6.7 (Supplementary Data 3).

#### Immunohistochemical detection of AgRP, POMC, and KP neurons

The RNA-preserving immunohistochemical protocol optimized for cortical parvalbumin labeling was adapted to detect hypothalamic AgRP, POMC, and KP neurons. All antibody and buffer solutions were supplemented with 2% PVSA, except the epitope retrieval solution, which contained 10% PVSA, and the peroxidase developer, which was free of RNase inhibitors.

Mediobasal hypothalamic sections (every 12th) were removed from the cryoprotectant solution, rinsed in ice-cold PBS (3 × 5 min), subjected to antigen retrieval in 0.1 M citrate buffer (pH 6.0; 80 °C; 30 min), then incubated at 4 °C for 20 min in PBS containing 1% H_2_O_2_ and 0.5% Triton X-100. The following primary antibodies were applied for 40 h at 4 °C: goat anti-AgRP (#EB06725, G2; Everest Biotech; 1:10,000); sheep anti-POMC (#41; gift from Dr. Csaba Fekete; 1:30,000)^42^; sheep anti-KP-54 (GQ2; gift from Dr. Waljit S. Dhillo; 1:50,000)^43^. After PBS washes, sections were incubated for 2 h at RT with species-specific biotinylated secondary antibodies raised in donkeys (Jackson ImmunoResearch; 1:500), followed by a 1-h incubation with the ABC Elite reagent (Vector Laboratories, Burlingame, CA, USA; 1:1000) in PBS. Immunoreactivity was visualized using Ni-DAB chromogen as described for cortical parvalbumin labeling. For reagents, see Supplementary Data 8.

#### LCM

Immunolabeled sections were mounted onto PEN membrane glass slides (Arcturus PEN membrane glass slides, Thermo Fisher Scientific) from Elvanol containing 2% PVSA, air-dried, and processed for LCM via sequential dehydration: 70% ethanol (60 s), 96% ethanol (60 s), 100% ethanol (2 × 60 s), and xylene (60 s). Slides were stored at −80 °C in slide mailers with silica gel desiccants unless processed immediately for LCM. Using a PALM Microbeam system (Carl Zeiss) equipped with a ×40 objective lens and PALMRobo 4.9 Pro Software, immunolabeled neurons were individually selected from the INF and pressure-catapulted into adhesive caps (Adhesive Cap 500, Carl Zeiss). Approximately 850 neurons were collected per sample and stored at −80 °C in LCM caps until RNA extraction.

#### RNA extraction

Total RNA was isolated from pooled neurons using the Arcturus Paradise PLUS FFPE RNA Isolation Kit (Applied Biosystems, Waltham, MA, USA), according to the manufacturer’s protocol.

#### Library preparation and RNA sequencing

Sequencing libraries were generated with the TruSeq Stranded Total RNA Library Preparation Gold kit (Illumina, San Diego, CA, USA), incorporating 16 PCR cycles for fragment enrichment^6,44^. A pooled library mix (1 nM; 120 µl total volume) containing 8 indexed samples was sequenced on an Illumina NextSeq 500 platform using the NextSeq 500/550 High Output v2.5 kit (75-cycle configuration)^16^.

#### Bioinformatics

Low-quality reads and adapter sequences were removed using Trimmomatic (v0.39; parameters: LEADING:3, TRAILING:3, SLIDINGWINDOW:4:15, MINLEN:36)^45^ and Cutadapt (v4.6)^46^, respectively. Cleaned reads were aligned to the *Homo sapiens* GRCh38 Ensembl reference genome (release hs111)^47^ using the STAR aligner (v2.7.11b)^48^ with default parameters. Gene-level quantification was carried out using featureCounts (Subread v2.0.6)^49^. Transcriptome coverage was assessed based on the number of unique transcripts detected at two read count thresholds: ≥1 and ≥5 reads. Metrics were calculated and reported as the mean ± standard error of the mean (SEM) across biological replicates. Subsequent analysis was conducted in R (v4.5.0)^50^. TPM values were computed from read counts normalized to gene lengths obtained via FeatureCounts, using gene models defined in the corresponding Ensembl GTF annotation file. For differential expression analysis, raw gene counts were normalized and analyzed using the DESeq2 package (v1.44.0)^51^. Data processing, transformation, and visualization were performed in R using tidyverse (v2.0.0)^52^, ggplot2 (v3.5.2)^53^, cluster (v2.1.8.1)^54^, pheatmap (v1.0.12)^55^, EnhancedVolcano (v1.22.0)^56^, eulerr (v7.0.2)^57^, and fmsb (v0.7.6)^58^, with additional plotting and file-handling packages listed in the Code Availability statement.

For species comparisons, the mouse PRJNA281954 dataset on AgRP and POMC neurons of fed and fasted mice^14^ was reprocessed using the analysis pipeline described above. Reads were aligned to the GRCm39 reference genome with the corresponding Ensembl release 111 GTF annotation, and all downstream analyses were performed within the same workflow. For cross-species analyses, mouse Ensembl gene IDs were linked to their human orthologs using biomaRt with the Ensembl release 111 mouse mart.

#### Functional classification

Transcripts were assigned to functional categories using the KEGG BRITE database^9^ (https://www.genome.jp/kegg/brite.html). Transcriptional regulators were identified based on the KEGG ‘Transcription Factors’ category (hsa03000) (see Supplementary Data 6). Receptor classes (Supplementary Data 12) included G protein-coupled receptors (hsa04030), cytokine receptors (hsa04050), pattern recognition receptors (hsa04054), and nuclear receptors (hsa03310). This functional category was extended to include ACVR1C and INSR from the “receptor serine/threonine kinases” and “receptor tyrosine kinases” categories (hsa01001+hsa08873), respectively. Expression of receptor genes in a neuronal phenotype was defined by a mean TPM ≥ 10 and a minimum of 5 supporting reads in each biological replicate (Supplementary Data 12). Neuropeptides were classified based on the neuropeptides.nl database^10^ (Supplementary Data 7). lncRNAs expressed by AgRP, POMC, and KP neurons (Supplementary Data 9) were collected according to the biotype data from the Ensembl GTF annotation file (https://www.ensembl.org/info/genome/genebuild/biotypes.html).

#### Populational enrichment of gene expression

Gene expression was considered enriched in a specific neuronal phenotype if the transcript exhibited at least a twofold increase in normalized read counts (log_2_ FC ≥ 1) relative to the other populations. When two neuronal types each showed ≥twofold higher expression compared to the third, the gene was classified as co-enriched in those two phenotypes.

### Comparison of POMC subgroups in the dorsomedial and ventrolateral INF

POMC neurons were isolated by LCM from sections corresponding to plate 27 of the Mai et al. human brain atlas^15^, where dorsomedial (DM) and ventrolateral (VL) POMC-IR cell groups could be most clearly separated. Analysis was performed using sections from two donors, aged 28 and 57 years. (Samples #6 and #9; Supplementary Data 3). Sequencing and bioinformatic processing were performed as described above, and transcript abundance was quantified as both read counts and TPM (Supplementary Data 16). Transcripts with ≥5 reads and TPM ≥ 10 in the same subnucleus of both specimens were analyzed for regional enrichment. Receptor genes were subjected to additional effect-size filtering and visualized in Fig. 9c if they showed an absolute DM–VL log_2_ FC > 0.2 in both individuals and a mean log_2_ FC > 0.5 across individuals.

### GWAS associations

Trait- and disease-associated SNPs were retrieved from the GWAS Catalog^13^ (https://www.ebi.ac.uk/gwas/). Traits and diseases relevant to malnutrition were manually curated and assigned to the 14 mapped GWAS traits presented in Table 1 and Supplementary Data 10. Traits included all descendant terms, except for the “Body weight” category. For comparative analysis, only intragenic SNPs reaching genome-wide significance (*P* < 5 × 10^−8^) were retained. The resulting gene list was then cross-referenced with neuronal population-specific enrichment data (Supplementary Data 11 and 12).

### Quantification and statistical analysis

Statistical analyses for Fig. 1d were conducted using one-way ANOVA followed by Tukey’s HSD post hoc tests, implemented via the aov() and TukeyHSD() functions in R. Normality was evaluated with the Shapiro–Wilk test, and homogeneity of variances was assessed using Levene’s test (Brown–Forsythe; median-centered), via the shapiro.test() and leveneTest() functions. Significance was set at *P* < 0.05. Statistical results are presented in Supplementary Data 2.

Sample sizes for the IHC/LCM-Seq experiments were determined by the availability of human specimens suitable for cell phenotype identification. Differential expression analysis for RNA-seq data was performed using the DESeq2 package with a negative binomial generalized linear model. *P* values were calculated using a two-sided Wald test and adjusted for multiple comparisons using the Benjamini–Hochberg false discovery rate method. Genes with *P*.adj. values < 0.05 were considered significantly differentially expressed. Detailed results are provided in Supplementary Data 5.

Significant SNP-trait associations from GWAS were defined by *P* < 5 × 10^−8^ (see refs. ^59,60^). The annotation and filtering of SNPs by intragenic localization and trait mapping are described above under GWAS Associations. Codes are available at https://github.com/goczbalazs/PRJNA1305226_1305334.

### Immunofluorescence experiments

#### Fixation and section preparation

Human hypothalamic tissue blocks from four male subjects (aged 35, 45, 49, and 50 years; Samples #10-13; Supplementary Data 3) were immersion-fixed in freshly prepared 4% formaldehyde in 0.1 M phosphate-buffered saline (PBS; pH 7.4) for 7–14 days at 4 °C. Blocks were then cryoprotected in 20% sucrose for 5 days at 4 °C, snap-frozen on powdered dry ice, and sectioned coronally at 30 µm using a Leica SM 2000R freezing microtome (Leica Microsystems). Sections were stored in cryoprotectant solution at −20 °C until further processing.

#### Fluorescent visualization of AgRP neurons and co-expressed peptides

Free-floating sections of the INF were thoroughly rinsed in PBS and incubated with a solution containing 1% H_2_O_2_ and 0.5% Triton X-100 for 20 min. Antigen retrieval was performed with 0.1 M citrate buffer (pH 6.0) at 80 °C for 30 min. Sections were then treated with Sudan Black^61^ to reduce lipofuscin-related autofluorescence and subsequently incubated with primary antibodies for 24 h at room temperature.

AgRP neurons were labeled using either guinea pig anti-AgRP antiserum (#GP-029-50, GP-029-10-201707-TK; Biosensis; RRID:AB_2492388; 1:20,000) or goat anti-AgRP antibody (#EB06725, G2; Everest Biotech; 1:10,000). Peptides co-expressed with AgRP were detected using the following primary antibodies: mouse anti-CART (CA61F4OP001; Gift of J.T. Clausen; 1:1000), goat anti-galanin (#EB09679; Everest Biotech; 1:2000), rabbit anti-NPY (#AAS25418C, QC19720-41593; Antibody Verify Inc., 1:1000), sheep anti-POMC (#41; gift of C. Fekete; 1:4000), rabbit anti-SP (IS-2/5; gift of P. Ciofi; 1:1000), and guinea pig anti-somatostatin (IS-3/51; gift of P. Ciofi; 1:1000). AgRP primary antibodies were reacted with biotin-conjugated secondary antibodies, followed by ABC Elite reagent (Vector Laboratories) and then, FITC-tyramide to amplify the signal (diluted 1:3000 with 0.05 M Tris-HCl buffer; pH 7.6, containing 0.003% H_2_O_2_; 15 min at room temperature). Primary antibodies against co-expressed peptides were reacted with Cy3- or Cy5-conjugated secondary antibodies (Jackson ImmunoResearch; 1:500; 2 h at room temperature). Labeled sections were mounted onto glass slides, coverslipped with Mowiol mounting medium, and analyzed with confocal microscopy. For immunohistochemical reagents, see also Supplementary Data 8.

#### Confocal microscopy

Fluorescent signals were acquired using a Zeiss LSM 900 confocal microscope (Carl Zeiss). High-resolution images were captured using 10×/0.45 NA, 20×/0.8 NA, and 63×/1.4 NA objectives, a 0.7–1× optical zoom, controlled by Zen software (Carl Zeiss; v3.10). Fluorophores were excited with the following laser lines: 488 nm for FITC, 561 nm for Cy3, and 640 nm for Cy5. Emission was detected at 500–540 nm for FITC, 570–620 nm for Cy3, 650–700 nm for Cy5. Spectral crosstalk between channels was minimized using the ZEN ’Smart Setup’ function. Confocal z-stacks were acquired with a z-step of 0.85-1 µm, pixel dwell time of 0.8–1.6 µs, image resolution of 1024 ×  1024 pixels, and a pinhole set to 1 Airy unit. Z-stacks were processed using maximum or average intensity projection in ImageJ. Final figures were assembled and adjusted for brightness and contrast in Adobe Photoshop.

### RNAscope in situ hybridization detection of CALCR mRNA in POMC neurons

Twenty-µm-thick free-floating sections including the INF region were prepared from the hypothalamus of a 28-year-old male subject (Sample #6; Supplementary Data 3) with a freezing microtome. Sections were washed with RNase-free PBS (3×10 min), pretreated with 0.3% H_2_O_2_ (20 min), mounted onto Superfrost Ultra Plus slides (Epredia™, Product Code: 11976299), air-dried, baked at 60 °C (1 h), and finally stored at –20 °C. Prior to hybridization, boxes were equilibrated to room temperature to avoid humid precipitation. Sections were then processed according to the manufacturer’s protocol (RNAscope® Multiplex Fluorescent Reagent Kit v2, Document Number 323100-USM) with slight modifications introduced by Kormos et al.^62^ for optimizing concurrent mRNA and protein detection.

For *CALCR* mRNA detection, Hs-CALCR probe (483041; ACD; Bio-Techne, Newark, CA, USA) was used, and signal detection was achieved using Opal 570 dye (1:1000 dilution in tyramide signal amplification buffer, REF: 322809). POMC neurons were visualized by immunofluorescent staining with sheep anti-POMC primary antibody (#41; gift from Dr. Csaba Fekete; 1:4000, overnight incubation), followed by a Cy5-conjugated anti-sheep secondary antibody (Jackson ImmunoResearch; 1:500, 3 h) at room temperature. To reduce lipofuscin-derived tissue autofluorescence, sections were treated with autofluorescence eliminator reagent (Merck/Millipore, Cat# 2160) following staining. Briefly, samples were washed in PBS (2 × 15 min), incubated with DAPI for 1 min, and washed again in PBS (5 min). This was followed by sequential dehydration in 30%, 50%, and 70% ethanol (5 min each). The eliminator reagent was then applied directly onto the sections for 30 s. Slides were subsequently washed in 70% ethanol (3 × 5 min) and then rinsed in PBS. Finally, sections were coverslipped using ProLong Glass Antifade Mountant (Invitrogen, Cat. no.: P36980).

Slide scanning was performed by Pannoramic MIDI II Digital Scanner (3DHISTECH Ltd., Budapest, Hungary), using a 20×/0.8 NA Plan Apochromat objective (Zeiss), PCO.edge 4.2 back-illuminated sCMOS camera, and DAPI-Q, SR_LF635, and Cy3.5 SB filters. High-resolution digital images were exported from SlideViewer (v2.9.0.229983) and adjusted for brightness and contrast in ImageJ (v1.54p).

### Reporting summary

Further information on research design is available in the Nature Portfolio Reporting Summary linked to this article.

## Supplementary information

Peer Review file

Description of Additional Supplementary Files

Supplementary Data 1

Supplementary Data 2

Supplementary Data 3

Supplementary Data 4

Supplementary Data 5

Supplementary Data 6

Supplementary Data 7

Supplementary Data 8

Supplementary Data 9

Supplementary Data 10

Supplementary Data 11

Supplementary Data 12

Supplementary Data 13

Supplementary Data 14

Supplementary Data 15

Supplementary Data 16

Reporting Summary

## Data Availability

The human RNA-seq data generated in this study have been deposited in the NCBI Sequence Read Archive under the BioProject accession codes PRJNA1305226 for AgRP- and POMC-IR neurons, PRJNA1453244 for dorsomedial and ventrolateral POMC-IR neurons, and PRJNA1305334 for KP-IR neurons. Whole-transcriptome profiles of human AgRP-, POMC-, and KP-IR neurons generated in this study, including raw counts, TPM values, and DESeq2-derived log_2_ fold change and FDR statistics, are provided in Supplementary Data 5. Whole-transcriptome profiles of dorsomedial and ventrolateral POMC-IR neurons, including raw counts, TPM values, and log_2_ fold change, are provided in Supplementary Data 16. The publicly available murine RNA-seq data re-analyzed in this study are available in the NCBI Sequence Read Archive under the BioProject accession code PRJNA281954.

## Code availability

Analyses were performed in R (v4.5.0). The main packages used were DESeq2 (v1.44.0), tidyverse (v2.0.0), ggplot2 (v3.5.2), cluster (v2.1.8.1), pheatmap (v1.0.12), EnhancedVolcano (v1.22.0), eulerr (v7.0.2), and fmsb (v0.7.6). Additional packages used for file handling and graphical formatting included openxlsx (v4.2.8), ggfortify (v0.4.17), RColorBrewer (v1.1-3), ggrepel (v0.9.6), gridExtra (v2.3), ggpubr (v0.6.0), and scales (v1.4.0). Custom scripts are available at https://github.com/goczbalazs/PRJNA1305226_1305334.
