# Supplementary material for: Transcriptome profiling of human hypothalamic agouti-related protein and proopiomelanocortin neurons regulating energy homeostasis
Source: Nat Commun. 2026 Aug 15;17:9801. doi: 10.1038/s41467-026-76688-w (PMC13575109; doi:10.1038/s41467-026-76688-w)
Supplement: Supplementary file 1 [file 41467_2026_76688_MOESM19_ESM.pdf]

nature portfolio

Corresponding author(s): Szabolcs Takács, Erik Hrabovszky

Last updated by author(s): Jul 17, 2026

Reporting Summary

Nature Portfolio wishes to improve the reproducibility of the work that we publish. This form provides structure for consistency and transparency in reporting. For further information on Nature Portfolio policies, see our Editorial Policies and the Editorial Policy Checklist.

Statistics

For all statistical analyses, confirm that the following items are present in the figure legend, table legend, main text, or Methods section.

n/a	Confirmed	
☐	☒	The exact sample size (*n*) for each experimental group/condition, given as a discrete number and unit of measurement
☐	☒	A statement on whether measurements were taken from distinct samples or whether the same sample was measured repeatedly
☐	☒	The statistical test(s) used AND whether they are one- or two-sided<br>*Only common tests should be described solely by name; describe more complex techniques in the Methods section.*
☒	☐	A description of all covariates tested
☐	☒	A description of any assumptions or corrections, such as tests of normality and adjustment for multiple comparisons
☐	☒	A full description of the statistical parameters including central tendency (e.g. means) or other basic estimates (e.g. regression coefficient) AND variation (e.g. standard deviation) or associated estimates of uncertainty (e.g. confidence intervals)
☐	☒	For null hypothesis testing, the test statistic (e.g. F, t, r) with confidence intervals, effect sizes, degrees of freedom and P value noted<br>*Give P values as exact values whenever suitable.*
☒	☐	For Bayesian analysis, information on the choice of priors and Markov chain Monte Carlo settings
☒	☐	For hierarchical and complex designs, identification of the appropriate level for tests and full reporting of outcomes
☒	☐	Estimates of effect sizes (e.g. Cohen's *d*, Pearson's *r*), indicating how they were calculated

Our web collection on statistics for biologists contains articles on many of the points above.

Software and code

Policy information about availability of computer code

Data collection	RNA quality was assessed by determining RNA Integrity Numbers (RIN) using the Agilent 2100 Bioanalyzer system with 2100 Expert software (Agilent, Santa Clara, CA, USA; version B.02.10.SI764). Laser capture microdissection was performed using a PALM MicroBeam system (Carl Zeiss) equipped with a 40× objective lens and controlled by PALMRobo Pro software (version 4.9.0.0). RNA-seq libraries were sequenced on an Illumina NextSeq 500 platform using the NextSeq 500/550 High Output v2.5 kit in a 75-cycle configuration and controlled by Illumina NextSeq Control Software. Confocal microscopy was performed using a Zeiss LSM 900 confocal microscope mounted on an Axio Observer.z1/7 stand (Carl Zeiss). High-resolution images were acquired using 10×/0.45 NA, 20×/0.8 NA and 63×/1.4 NA objectives with 0.7–1× optical zoom and controlled by ZEN software (Carl Zeiss, version 3.10). Slide scanning was performed using a Pannoramic MIDI II Digital Scanner (3DHISTECH Ltd., Budapest, Hungary). High-resolution digital images were exported using SlideViewer software (version 2.9.0.229983) and adjusted for brightness and contrast in ImageJ (version 1.54p).
Data analysis	Low-quality reads and adapter sequences were removed using Trimmomatic (v0.39) and Cutadapt (v4.6). Cleaned reads were aligned to the Homo sapiens GRCh38 Ensembl reference genome (release hs111) using the STAR aligner (v2.7.11b). Gene-level quantification was carried out using featureCounts (Subread v2.0.6). Subsequent analysis was conducted in R (v4.5.0). TPM values were computed from read counts normalized to gene lengths obtained via featureCounts. For differential expression analysis, raw gene counts were normalized and analyzed using the DESeq2 package (v1.44.0). The following R packages were utilized for data transformation, analysis, and visualization: tidyverse (v2.0.0), openxlsx (v4.2.8), ggplot2 (v3.5.2), ggfortify (v0.4.17), cluster (v2.1.8.1), pheatmap (v1.0.12), RColorBrewer (v1.1-3), ggrepel (v0.9.6), EnhancedVolcano (v1.22.0), gridExtra (v2.3), ggpubr (v0.6.0), eulerr (v7.0.2), fmsb (v0.7.6), scales (v1.4.0). Custom scripts are available at https://github.com/goczbalazs/PRJNA1305226_1305334.

For manuscripts utilizing custom algorithms or software that are central to the research but not yet described in published literature, software must be made available to editors and reviewers. We strongly encourage code deposition in a community repository (e.g. GitHub). See the Nature Portfolio guidelines for submitting code & software for further information.

Data

Policy information about availability of data

All manuscripts must include a data availability statement. This statement should provide the following information, where applicable:

- Accession codes, unique identifiers, or web links for publicly available datasets
- A description of any restrictions on data availability
- For clinical datasets or third party data, please ensure that the statement adheres to our policy

Data were obtained from newly generated RNA-seq experiments performed in this study and from the re-analysis of publicly available raw RNA-seq datasets from murine AgRP and POMC neurons deposited under accession PRJNA281954. RNA-Seq files for human AgRP and POMC neurons are available in BioProject (https://www.ncbi.nlm.nih.gov/bioproject/) with the accession number PRJNA1305226 and PRJNA1453244, KP sequencing data have been deposited under PRJNA1305334.

Research involving human participants, their data, or biological material

Policy information about studies with human participants or human data. See also policy information about sex, gender (identity/presentation), and sexual orientation and race, ethnicity and racism.

Reporting on sex and gender	The overall tissue set comprised twelve male donors. In addition, tissue samples from one female brain was used for method optimization. Gender identity was not available from the de-identified records and was therefore not assessed; accordingly, biological sex rather than gender is reported. No sex-based comparative analyses were performed because the analytical dataset comprised only male donors.
Reporting on race, ethnicity, or other socially relevant groupings	Race/ethnicity information was obtained from autopsy administrative records and indicated that all donors were of European ancestry/ethnicity (Hungarian). This information is reported to describe cohort composition and to assist interpretation of the scope and generalizability of the findings. No analyses stratified by race or ethnicity were performed because the analytical dataset was homogeneous with respect to ancestry/ethnicity. Socioeconomic status was not collected because the samples were de-identified and these data were not available in the source records.
Population characteristics	Human postmortem brain tissue was obtained from adult donors during autopsies conducted at the Department of Pathology and Experimental Cancer Research, Semmelweis University, Budapest, Hungary. The available tissue cohort comprised thirteen adults with no documented neurological disorders in the clinical history, including cause-of-death records: one 53-year-old female and twelve male individuals aged 28, 33, 35, 38, 45, 46, 49, 50, 52, 53, 56 and 57 years. For IHC/LCM-Seq (n=9), tissue was collected within 12–24 h postmortem. This short postmortem interval was a key inclusion criterion.
Recruitment	Participants were not prospectively recruited for this study. Instead, de-identified postmortem human brain specimens were selected from consecutive available autopsies meeting the inclusion criteria at Semmelweis University. Inclusion criteria were availability of adult brain tissue, absence of documented neurological disorders in the clinical history, and postmortem interval of 24 h or less for IHC/LCM-Seq samples, with preference for the shortest available interval; no cases with a postmortem interval shorter than 12 h were available. The analytical transcriptomic dataset therefore comprised eight male donors meeting all of these criteria, whereas one female donor sample was used for method optimization only. Four male samples were used for dual-immunolabeling experiments.
Ethics oversight	Ethical approval for the use of human postmortem tissue was granted by the Regional and Institutional Committee of Science and Research Ethics of Semmelweis University (SE-TUKEB 251/2016). Tissue use complied with the Declaration of Helsinki and relevant Hungarian legislation, including Act CLIV of 1997 and Decree 18/1998 (XII.27.) of the Ministry of Health. Under the applicable Hungarian regulations, prior written consent from the deceased was not required for the use of this postmortem material.

Note that full information on the approval of the study protocol must also be provided in the manuscript.

Field-specific reporting

Please select the one below that is the best fit for your research. If you are not sure, read the appropriate sections before making your selection.

☒ Life sciences ☐ Behavioural & social sciences ☐ Ecological, evolutionary & environmental sciences

For a reference copy of the document with all sections, see nature.com/documents/nr-reporting-summary-flat.pdf

Life sciences study design

All studies must disclose on these points even when the disclosure is negative.

Sample size	Sample sizes for the IHC/LCM-Seq experiments were determined by the availability of human specimens suitable for cell phenotype identification. For methodological optimization, three experimental replicates were used for each parameter tested, using tissue from the same donor specimen. For phenotype-level transcriptomic profiling, approximately 850 immunoreactive neuronal perikarya were isolated by LCM for each bulk RNA-seq sample; profiling was performed on three AgRP-immunoreactive, two POMC-immunoreactive and three KP-immunoreactive neuronal samples. For the spatially resolved comparison of dorsomedial and ventrolateral POMC neurons within the INF, approximately 300–500 POMC-IR neurons were isolated per sample.

Data exclusions | No data were exluded from the analysis.

Replication | For methodological optimization of IHC/LCM-Seq, the baseline condition and each tested parameter setting were assessed in three experimental replicates using tissue from the same donor specimen. Results were consistent.
For phenotype-level IHC/LCM-Seq, transcriptome profiling was performed on independent postmortem human hypothalamic samples, including three AgRP-immunoreactive, two POMC-immunoreactive and three KP-immunoreactive neuronal samples. For each bulk RNA-seq sample, approximately 850 immunoreactive neuronal perikarya were isolated by LCM.
For the spatially resolved comparison of dorsomedial and ventrolateral POMC neurons within the INF, two donor samples were analyzed, with 300–500 POMC-immunoreactive neurons collected from each region in each subject.
For dual-immunofluorescent validation of neuropeptide co-expression, two independent experiments, each using two different biological samples yielded consistent results.
For the combined IHC and RNAscope experiment, following successful immunostaining and probe hybridization, co-expression was quantified in three tissue sections from a single donor, with highly consistent numbers of single- and double-labelled POMC cells and co-expression ratios across sections.

Randomization | Randomization was not applicable because the study involved transcriptomic analysis of predefined neuronal populations in postmortem human hypothalamic specimens. No treatments or experimental conditions were assigned to subjects or samples.

Blinding | Blinding was not applicable because neuronal populations and regions of interest were predefined by immunoreactivity, marker identity, and anatomical location, which had to remain visible during LCM and imaging analyses. No subjective group allocation was performed, and sequencing data were analyzed using predefined bioinformatic and statistical pipelines.

Reporting for specific materials, systems and methods

We require information from authors about some types of materials, experimental systems and methods used in many studies. Here, indicate whether each material, system or method listed is relevant to your study. If you are not sure if a list item applies to your research, read the appropriate section before selecting a response.

Materials & experimental systems

n/a	Involved in the study	
☐	☒	Antibodies
☒	☐	Eukaryotic cell lines
☒	☐	Palaeontology and archaeology
☒	☐	Animals and other organisms
☒	☐	Clinical data
☒	☐	Dual use research of concern
☒	☐	Plants

Methods

n/a	Involved in the study	
☒	☐	ChIP-seq
☒	☐	Flow cytometry
☒	☐	MRI-based neuroimaging

Antibodies

Antibodies used | The following primary antibodies were used:
Goat anti-AgRP (1:10,000; EB06725 (LOT# G2); Everest Biotech),
Guinea pig anti-AgRP (1:20,000; GP-029-50 (LOT# GP-029-10-201707-TK); Biosensis),
Mouse anti-CART (1:1,000; CA61F4OP001; Gift of J.T. Clausen),
Goat anti-GAL (1:2,000; EB09679; Everest Biotech),
Sheep anti-KP-54 (1:50,000; GQ2; gift from W.S. Dhillo),
Rabbit anti-NPY (1:1,000; AAS25418C (LOT# QC19720-41593); Antibody Verify Inc.),
Sheep anti-POMC (1:4,000 (IF) and 1:30,000 (with DAB chromogen); #41; gift from Dr. Csaba Fekete),
Goat anti-PVALB (1:30,000; EB06776 (LOT# P1); Everest Biotech),
Rabbit anti-SP (1:1,000; IS-2/5; gift from P. Ciofi),
Guinea pig anti-SST (1:1,000; IS-3/51; gift from P. Ciofi).

The following secondary antibodies were used from Jackson ImmunoResearch (in a dilution of 1:500) :
Biotin-SP-AffiniPure donkey anti-guinea pig IgG (H+L; #706-065-148),
Biotin-SP-AffiniPure donkey anti-goat IgG (H+L; #705-065-147),
Biotin-SP-AffiniPure donkey anti-sheep IgG (H+L; #713-065-147),
Cy3 AffiniPure donkey anti-mouse IgG (H+L; #715-165-150),
Cy3 AffiniPure donkey anti-goat IgG (H+L; #705-165-147),
Cy3 AffiniPure donkey anti-guinea pig IgG (H+L; #706-165-148),
Cy3 AffiniPure donkey anti-rabbit IgG (H+L; #711-165-152),
Cy5 AffiniPure donkey anti-sheep IgG (H+L; #713-175-147).

See in details in Supplementary Table 8.

Validation | Relevant citations for specificity validation:
doi: 10.1371/journal.pone.0103977 for EB06725, GP-029-50 and CA61F4OP001

doi: 10.3389/fnins.2015.00029 for EB09679 and IS-3/51
doi: 10.1007/s00429-017-1553-5 for #41
doi: 10.1210/jc.2005-1468 for GQ2
doi: 10.1016/0143-4179(88)90039-X for IS-2/5
doi: 10.1016/j.neuroscience.2006.05.041 for IS-3/51

Plants

Seed stocks	*Report on the source of all seed stocks or other plant material used. If applicable, state the seed stock centre and catalogue number. If plant specimens were collected from the field, describe the collection location, date and sampling procedures.*
Novel plant genotypes	*Describe the methods by which all novel plant genotypes were produced. This includes those generated by transgenic approaches, gene editing, chemical/radiation-based mutagenesis and hybridization. For transgenic lines, describe the transformation method, the number of independent lines analyzed and the generation upon which experiments were performed. For gene-edited lines, describe the editor used, the endogenous sequence targeted for editing, the targeting guide RNA sequence (if applicable) and how the editor was applied.*
Authentication	*Describe any authentication procedures for each seed stock used or novel genotype generated. Describe any experiments used to assess the effect of a mutation and, where applicable, how potential secondary effects (e.g. second site T-DNA insertions, mosiacism, off-target gene editing) were examined.*
