# Supplementary material for: Transcriptome profiling of human hypothalamic agouti-related protein and proopiomelanocortin neurons regulating energy homeostasis
Source: Nat Commun. 2026 Aug 15;17:9801. doi: 10.1038/s41467-026-76688-w (PMC13575109; doi:10.1038/s41467-026-76688-w)
Supplement: Supplementary file 2 [file 41467_2026_76688_MOESM1_ESM.pdf]

nature portfolio

Peer Review File

Transcriptome profiling of human hypothalamic agouti-related protein and proopiomelanocortin neurons regulating energy homeostasis

Corresponding Author: Dr Erik Hrabovszky

This file contains all reviewer reports in order by version, followed by all author rebuttals in order by version.

Version 0:

Reviewer comments:

Reviewer #1

(Remarks to the Author)
In this manuscript, Takács and colleagues have developed a new technique, IHC/LCM, which they use to identify and capture POMC, AGRP and KISS neurons from postmortem human hypothalami, and perform downstream bulk transcriptomic analyses.

There are elements to this work that are novel and exciting, but others less so.

The novel elements are the IHC/LCM technique they have developed, which allow for identification of proteins in specific neurons, yet preserves the RNA for transcriptional analyses. The second element is their approach also allows the identification and characterization of lncRNAs.

The major issue I have with this is the bulk RNAseq of these neurons, after their painstaking individual identification and extraction. The authors call these neuronal 'populations', but in reality, because of the known underlying heterogeneity in the three types of neurons they have studied, they only provide an average expression profile. Just taking the POMC and AGRP neurons as an example, the authors discuss their expression of receptors GLP1R and INSR. But multiple groups have already shown that not these receptors are only expressed on a subset of the two groups of neurons in question. This is true for most of the transcripts they have highlighted as enriched in the neurons they are studying.

Given their new technique, I think that the authors need to take a number of top candidates, and go back to their brain slices and overlap the IHC with in situ hybridization to both validate their findings, and more importantly, to illustrate the heterogeneity of the POMC, AGRP and KISS neurons, but in a spatial context.

(Remarks on code availability)

Reviewer #2

(Remarks to the Author)
This paper by Takacs, et al. reports a method for combining laser capture microdissection (LCM) with immunohistochemistry (IHC) in human postmortem brain tissue to extract mRNA from molecularly defined neuron populations. They report differential gene expression analysis for human POMC, AGRP, and KISS neurons. These neurons have been studied extensively in mice for their role in obesity and reproduction. The methods description is straightforward and the gene expression analysis is interesting. The major issue is that the authors did not make sufficient attempts to validate their results against previously established co-expression relationships from public data. Overall, there needs to be greater emphasis on validation of their reported results against available mouse and human datasets for the same neurons.

1. There are discrepancies in human and mouse gene expression data between mouse and human. For example, in cell type specific bulk RNA_Seq (PMID 26329458), which is similar to the approach used here, Cartpt is reported found in mice to be predominantly associated with POMC neurons over AGRP neurons, but Fig 4a reports the opposite. Similarly Calcr is

reported to be primarily expressed in AGRP neurons in mice but the authors indicate that it is in POMC neurons
2. There is not an attempt to reconcile the co-expression relationships reported in using their method with expectations that would be from the human Hypomap data.

(Remarks on code availability)

Reviewer #3

(Remarks to the Author)
This study achieves deep transcriptomic profiling of human hypothalamic AgRP, POMC, and KP neurons through the innovative IHC/LCM-Seq technique. The authors demonstrate that this method is applicable to brain tissues collected 24 hours postmortem and can detect more transcripts compared to single-nucleus sequencing. Using this approach, the authors systematically analyzed the transcriptome profiling of these three neuronal populations in the human brain, revealing significant expression differences—particularly in neuropeptides, metabolic-related genes, and receptors. Overall, this research recharacterizes the transcriptome profiling of human POMC, AgRP, and KP neurons with a relatively novel methodology, providing abundant additional insights and serving as a valuable resource for the field.

Below are some points to address for the improvement of the manuscript:
1. More discussions are needed regarding the differences between the findings of this study and those obtained using single-nucleus sequencing technology (PMID: 39910307).
2. As the authors stated, the IHC/LCM-Seq technique preserves the spatial context of neurons, yet this technical advantage is not fully demonstrated in the current study. POMC neurons exhibit high heterogeneity and a relatively dispersed distribution within the arcuate nucleus (ARC). Could the authors analyze transcriptomic differences in POMC neurons located in distinct regions (e.g., medial vs. lateral ARC)? Such data would better highlight the technical strengths of IHC/LCM-Seq and provide more comprehensive insights.
3. This study employs bulk sequencing rather than single-cell sequencing, which precludes the resolution of neuronal subpopulation heterogeneity (e.g., previous studies have identified multiple subclusters of human POMC neurons). The authors should at least discuss this limitation in the manuscript.

(Remarks on code availability)

Version 1:

Reviewer comments:

Reviewer #1

(Remarks to the Author)
The authors have satisfactorily responded to my comments and critique.

(Remarks on code availability)

Reviewer #2

(Remarks to the Author)
The authors have addressed my concerns and the manuscript is suitable for publication.

(Remarks on code availability)

Reviewer #3

(Remarks to the Author)
All my concerns have now been fully addressed. I therefore support its publication in Nature Communications.

(Remarks on code availability)

Open Access This Peer Review File is licensed under a Creative Commons Attribution 4.0 International License, which permits use, sharing, adaptation, distribution and reproduction in any medium or format, as long as you give appropriate credit to the original author(s) and the source, provide a link to the Creative Commons license, and indicate if changes were made.
In cases where reviewers are anonymous, credit should be given to 'Anonymous Referee' and the source.
The images or other third party material in this Peer Review File are included in the article's Creative Commons license, unless indicated otherwise in a credit line to the material. If material is not included in the article's Creative Commons license and your intended use is not permitted by statutory regulation or exceeds the permitted use, you will need to obtain permission directly from the copyright holder.
To view a copy of this license, visit https://creativecommons.org/licenses/by/4.0/

REVIEWER COMMENTS

Reviewer #1 (Remarks to the Author):

In this manuscript, Takács and colleagues have developed a new technique, IHC/LCM, which they use to identify and capture POMC, AGRP and KISS neurons from postmortem human hypothalami, and perform downstream bulk transcriptomic analyses.

There are elements to this work that are novel and exciting, but others less so.

The novel elements are the IHC/LCM technique they have developed, which allow for identification of proteins in specific neurons, yet preserves the RNA for transcriptional analyses. The second element is their approach also allows the identification and characterization of lncRNAs.

The major issue I have with this is the bulk RNAseq of these neurons, after their painstaking individual identification and extraction. The authors call these neuronal 'populations', but in reality, because of the known underlying heterogeneity in the three types of neurons they have studied, they only provide an average expression profile. Just taking the POMC and AGRP neurons as an example, the authors discuss their expression of receptors GLP1R and INSR. But multiple groups have already shown that not these receptors are only expressed on a subset of the two groups of neurons in question. This is true for most of the transcripts they have highlighted as enriched in the neurons they are studying.

Given their new technique, I think that the authors need to take a number of top candidates, and go back to their brain slices and overlap the IHC with in situ hybridization to both validate their findings, and more importantly, to illustrate the heterogeneity of the POMC, AGRP and KISS neurons, but in a spatial context.

Responses to Reviewer 1

We thank the Reviewer for the thoughtful critique and specific suggestions, which have substantially improved the revised manuscript. We addressed the concerns regarding the existence and heterogeneity of neuronal subgroups, the validation of gene expression data by IHC and ISH, and the spatial context of the transcriptomic findings as follows:

i) We acknowledge that bulk sequencing provides composite information from multiple AgRP-, POMC-, and KP-immunoreactive neuronal subgroups. Accordingly, we have revised the text to avoid referring to these neurons as "populations."

ii) The important issue of neuronal heterogeneity is discussed in a new methodological paragraph (Line 309: …"We note that achieving this level of transcriptome coverage involves a trade-off in cellular resolution"…).

iii) We carefully examined the recent human HYPOMAP study, in which the authors identified a single AgRP neuron cluster and proposed that POMC neurons may comprise three subclusters defined by signature receptor transcripts, particularly *CALCR* mRNA, which encodes the

calcitonin receptor. To assess the topographic distribution of *CALCR* mRNA among POMC-immunoreactive neurons, we performed RNAscope in situ hybridization experiments. These new analyses confirmed that not all POMC neurons express this receptor. The proportion of *CALCR*-expressing POMC-immunoreactive neurons tended to be higher in the dorsomedial (~21%) than in the ventrolateral (~12%) infundibular nucleus. *CALCR*-positive and *CALCR*-negative POMC neurons were intermingled in both subdivisions. These new findings are now presented in the new Results section (…"Spatial context shapes the transcriptome of human POMC neurons"…; line 231), illustrated in new Figures 9a and 9b, and discussed in detail in a new Discussion section (…"heterogeneity among POMC-immunoreactive neurons necessarily remained unexplored in our bulk datasets"…; line 311).

iv) POMC neurons in our RNAscope study formed two anatomically distinguishable subgroups that differed in the extent of *CALCR* co-expression. To address additional spatial heterogeneities, we designed and carried out new IHC/RNA-Seq experiments on these two POMC-immunoreactive neuronal groups. This new bulk sequencing study confirmed mediolateral heterogeneity of POMC neurons in the expression of *CALCR* and many other transcripts, demonstrated the spatial power of IHC/RNA-Seq. These new findings are described in the new Results section (Line 244: …"To capitalize on the high spatial resolution of the IHC/LCM-Seq method, we further analyzed the anatomically distinct DM and VL subpopulations of POMC neurons"…) and illustrated in Fig. 9c.

v) Although the selective detection of *POMC*, *AGRP*, and *KISS1* marker mRNAs (Fig. 2f) confirms that the three transcriptomes were derived from three non-overlapping neuronal phenotypes, we agreed to validate selected RNA-Seq findings by immunohistochemistry. Our new dual- and triple-immunofluorescence experiments confirmed the coexpression of *NPY*, *CARTPT*, *SST*, *GAL*, and *TAC1* (encoding substance P) in subsets of AgRP-immunoreactive neurons, validating our IHC/LCM-Seq data. The new results (Line 160:… „Immunofluorescence experiments validating some of these observations"…) are illustrated in the new Fig. 5. We note that AgRP neurons forming only a single cluster in the HYPOMAP study showed considerable heterogeneity in their neuropeptide immunoreactivities.

We thank the Reviewer again for the careful evaluation of our manuscript and for the constructive comments that helped us improve both the scope and the clarity of the study.

Reviewer #2 (Remarks to the Author):

This paper by Takacs, et al. reports a method for combining laser capture microdissection (LCM) with immunohistochemistry (IHC) in human postmortem brain tissue to extract mRNA from molecularly defined neuron populations. They report differential gene expression analysis for human POMC, AGRP, and KISS neurons. These neurons have been studied extensively in mice for their role in obesity and reproduction. The methods description is straightforward and the gene expression analysis is interesting. The major issue is that the authors did not make sufficient attempts to validate their results against previously established co-expression relationships from public data. Overall, there needs to be greater emphasis on validation of their reported results against available mouse and human datasets for the same neurons.

1. There are discrepancies in human and mouse gene expression data between mouse and human. For example, in cell type specific bulk RNA_Seq (PMID 26329458), which is similar to the approach used here, Cartpt is reported found in mice to be predominantly associated with POMC neurons over AGRP neurons, but Fig 4a reports the opposite. Similarly Calcr is reported to be primarily expressed in AGRP neurons in mice but the authors indicate that it is in POMC neurons (IHC)
2. There is not an attempt to reconcile the co-expression relationships reported in using their method with expectations that would be from the human Hypomap data.

Responses to Reviewer 2

We would like to thank the Reviewer for the helpful and constructive comments on our manuscript. In response, we have carried out new experiments and revised the manuscript extensively to address the specific points.

'1. There are discrepancies in human and mouse gene expression data between mouse and human. For example, in cell type specific bulk RNA_Seq (PMID 26329458), which is similar to the approach used here, Cartpt is reported found in mice to be predominantly associated with POMC neurons over AGRP neurons, but Fig 4a reports the opposite. Similarly[HE3.1] Calcr is reported to be primarily expressed in AGRP neurons in mice but the authors indicate that it is in POMC neurons (IHC)'

Thank you very much for the suggestion to more clearly highlight species differences between rodents and humans. We have addressed this issue in several ways in the revised manuscript:

Indeed, the presence of CART peptide in human AgRP neurons, but not in POMC neurons, represents an important difference from the rodent condition. This observation was originally reported by Csaba Fekete's group, whose contribution we acknowledge by citing their work as Ref. 11.

We also present new immunohistochemical data confirming the presence of CART protein in human AgRP neurons, but not in POMC neurons (Fig. 5c). This observation is in agreement with

our transcriptomic results illustrated in Fig. 4. We also mention and cite our earlier immunohistochemical finding of CART in human KP neurons (Ref. 21), another species difference from rodents.

To address more thoroughly species differences, we carried out a meta-analysis of the murine AgRP and POMC neuron transcriptomes from PMID 26329458 (Ref. 14). Neuropeptides expressed at TPM ≥ 40 and the top 40 receptor transcripts (including potential drug targets) expressed in AgRP and POMC neurons of either humans or mice are now presented in a new Results section in Line 208 (…"Neuropeptide and receptor profiles differ markedly in humans and mice"…) and these differences are illustrated in Figs. 8a-c. As the Reviewer noted, the pattern of *Calcr/CALCR* co-expression differs between the two species, being detectable in human POMC neurons and in mouse AgRP neurons (Fig. 8c).

The revised manuscript now also includes new RNAscope in situ hybridization results (Figs. 9a andb) and new IHC/LCM-Seq data (Fig. 9c), both of which confirm *CALCR* expression in the human POMC neuron phenotype.

Species differences have been given greater emphasis in the revised Discussion, including in Lines 338 ("This pattern highlights a notable species difference…") and 391 ("Our study identified similarities as well as robust species differences…").

2. There is not an attempt to reconcile the co-expression relationships reported in using their method with expectations that would be from the human Hypomap data.

Thank you very much for drawing our attention to the missing comparative aspect of bulk versus single-cell transcriptomics, which was also raised by the other two reviewers. Because the human HYPOMAP study identified only a single AgRP neuron cluster, we focused our attention on the POMC neuronal phenotype, which that study proposed to comprise three subclusters distinguished by signature receptor transcripts, particularly *CALCR*. We therefore first used RNAscope in situ hybridization to examine the topographic distribution of *CALCR* expression in POMC-immunoreactive neurons separately in the ventromedial and dorsolateral subdivisions of the INF. The results of this new experiment showed preferential enrichment of this receptor in the dorsomedial subdivision of the INF, consistent with the observations of the HYPOMAP study. At the same time, our results also showed that *CALCR*-positive POMC neurons represent only a minority of all POMC neurons (~15%) and are intermingled with *CALCR*-negative POMC neurons throughout the INF (Figs. 9a, b).

As a second approach to distinguishing between potential POMC neuron subtypes, we performed spatial transcriptomic analysis using IHC/LCM-Seq on dorsomedially versus ventrolaterally located POMC-immunoreactive neurons from two hypothalami. These new experiments revealed considerable transcriptomic differences between the two locations. (We note that these differences could not be mapped unambiguously onto any two of the three proposed POMC neuron subclusters defined in HYPOMAP.)

The new RNAscope and IHC/LCM-Seq Results are reported in Line 231 (subheading: 'Spatial context shapes the transcriptome of human POMC neurons' and illustrated in Figs. 9a-c.

Two newly added paragraphs in Line 294 (…"We propose that IHC/LCM-Seq and single-cell approaches are complementary methodologies"…) discuss abundantly methodological differences and POMC neuron heterogeneity.

We would like to thank the Reviewer once again for the careful evaluation of our manuscript and for the constructive comments.

Reviewer #3 (Remarks to the Author):

This study achieves deep transcriptomic profiling of human hypothalamic AgRP, POMC, and KP neurons through the innovative IHC/LCM-Seq technique. The authors demonstrate that this method is applicable to brain tissues collected 24 hours postmortem and can detect more transcripts compared to single-nucleus sequencing. Using this approach, the authors systematically analyzed the transcriptome profiling of these three neuronal populations in the human brain, revealing significant expression differences—particularly in neuropeptides, metabolic-related genes, and receptors. Overall, this research recharacterizes the transcriptome profiling of human POMC, AgRP, and KP neurons with a relatively novel methodology, providing abundant additional insights and serving as a valuable resource for the field.

Responses to Reviewer 3

Below are some points to address for the improvement of the manuscript:
1. More discussions are needed regarding the differences between the findings of this study and those obtained using single-nucleus sequencing technology (PMID: 39910307).

We thank the Reviewer for highlighting the importance of placing our bulk transcriptomic data in the context of recently published single-cell studies. The expanded Discussion now includes a new paragraph emphasizing the technical differences between single-cell approaches and our bulk sequencing method (Line 294: "We propose that IHC/LCM-Seq and single-cell approaches are complementary methodologies…"). For comparison with our work, the revised manuscript refers to the HYPOMAP study in Lines 59, 233, 244, 253, 312, 323, 339, and 342.

2. As the authors stated, the IHC/LCM-Seq technique preserves the spatial context of neurons, yet this technical advantage is not fully demonstrated in the current study. POMC neurons exhibit high heterogeneity and a relatively dispersed distribution within the arcuate nucleus (ARC). Could the authors analyze transcriptomic differences in POMC neurons located in distinct regions (e.g., medial vs. lateral ARC)? Such data would better highlight the technical strengths of IHC/LCM-Seq and provide more comprehensive insights.

Thank you very much for this valuable suggestion. As requested, and to address similar criticism raised by Reviewer 2, we attempted to leverage the spatial resolution of IHC/LCM-Seq to identify regional heterogeneity and potential subtypes within the POMC neuronal system. The HYPOMAP study suggested that POMC neurons comprise three subclusters distinguished by signature receptor transcripts, particularly *CALCR*. We therefore first used RNAscope *in situ* hybridization to examine the topographic distribution of *CALCR* expression in POMC-immunoreactive neurons separately in the ventromedial and dorsolateral subdivisions of the INF. The results of this new experiment (Line 231: …"Spatial context shapes the transcriptome of human POMC neurons"…) showed preferential enrichment of this receptor in the dorsomedial subdivision of the INF, consistent with the observations of the HYPOMAP study. At the same time, our results also showed that *CALCR*-positive POMC neurons represent only a minority of all POMC neurons (~15%) and are intermingled with *CALCR*-negative POMC neurons throughout the INF. These data are illustrated in Figs. 9a and b. As a second approach to distinguishing between potential

POMC neuron subtypes, we performed spatial transcriptomic analysis using IHC/LCM-Seq on dorsomedially versus ventrolaterally located POMC-immunoreactive neurons using two hypothalami. These new experiments revealed considerable transcriptomic differences between the two locations. We note that these differences could not be mapped unambiguously onto any two of the three proposed POMC neuron subclusters defined in HYPOMAP. Furthermore, we note that, whereas the HYPOMAP studies defined only a single type of AgRP neuron, our newly added immunofluorescence studies provided evidence for varying degrees of neuropeptide coexpression within AgRP neurons (Fig. 8).

3. This study employs bulk sequencing rather than single-cell sequencing, which precludes the resolution of neuronal subpopulation heterogeneity (e.g., previous studies have identified multiple subclusters of human POMC neurons). The authors should at least discuss this limitation in the manuscript.

Thank you very much for this suggestion as well. In addition to discussing the general advantages and limitations of bulk sequencing compared with single-cell sequencing (Line 294: “We propose that IHC/LCM-Seq and single-cell approaches are complementary methodologies…”), the revised manuscript now includes new immunofluorescence (Fig. 5), RNAscope (Figs. 9a and b), and spatial IHC/LCM-Seq (Fig. 9c) experiments, all of which address and demonstrate neuronal heterogeneity. Multiple comparisons with the HYPOMAP study are included both in the newly extended Results section (Line 231: “Spatial context shapes the transcriptome of human POMC neurons…”) and in the revised Discussion (Lines 309–328), addressing the important issue of neuronal heterogeneity within a single neuropeptide phenotype.

We would like to thank the Reviewer once again for the constructive evaluation of our work.
