# Supplementary material for: Transcriptome profiling of human hypothalamic agouti-related protein and proopiomelanocortin neurons regulating energy homeostasis
Source: Nat Commun. 2026 Aug 15;17:9801. doi: 10.1038/s41467-026-76688-w (PMC13575109; doi:10.1038/s41467-026-76688-w)
Supplement: Supplementary file 3 [file 41467_2026_76688_MOESM2_ESM.pdf]

Description of Additional Supplementary Files

Supplementary Data 1. Yield and integrity of RNA isolated from immersion-fixed human cortices

RIN values and RNA yield of samples isolated from immersion-fixed postmortem cortical brain blocks under different fixation time schedules (for Figure 1b).

Supplementary Data 2. RNA integrity after antigen retrieval and immunohistochemistry.

RIN values and one-way ANOVA results demonstrating RNA preservation during antigen retrieval and subsequent steps of the modified immunohistochemistry protocol (for Figure 1d).

Supplementary Data 3. Brain specimens used in different experiments

Postmortem human brain tissues used in different experiments.

Supplementary Data 4. Read and transcript counts in human AgRP, POMC, and KP neurons

Gene-assigned read counts and numbers of genes detected with ≥1 or ≥5 reads in each of the POMC, AgRP, and KP neuronal phenotypes (for Figures 2c, d).

Supplementary Data 5. Transcriptome profiling of human AgRP, POMC, and KP neurons

Whole-transcriptome profiles of hypothalamic AgRP, POMC, and KP neurons, including TPM values and DESeq2-derived log2 fold change and FDR statistics (for Figures 2e, f and Figures 3a-e).

Supplementary Data 6. Transcription factors expressed in human AgRP, POMC, and KP neurons

Gene expression profiles of transcription factors classified according to the KEGG BRITE category 'Transcription Factors' (ko03000; for Figures 3f, g).

Supplementary Data 7. Neuropeptides and granins expressed in human AgRP, POMC, and KP neurons

Gene expression profile of neuropeptides and granins curated from the Neuropeptide Database (www.neuropeptides.nl; for Figure 4).

Supplementary Data 8. Antibodies and immunohistochemical reagents

Antibodies and reagents used in immunohistochemical and immunofluorescence experiments

Supplementary Data 9. lncRNAs in human AgRP, POMC, and KP neurons

lncRNA expression profiles of AgRP, POMC, and KP neurons based on biotype annotations from the Ensembl GTF file.

Supplementary Data 10. Association data for metabolism-related GWAS traits

Association data for metabolism-related GWAS Catalog mapped traits (https://www.ebi.ac.uk/gwas/; for Table 1 and Figure 6).

Supplementary Data 11. Transcripts associated with metabolic disorders in human AgRP, POMC, and KP neurons

Transcripts of GWAS Catalog MAPPED_GENEs associated with metabolic disorders and expressed in AgRP, POMC, or KP neurons (for Figure 6).

Supplementary Data 12. Receptors expressed in human AgRP, POMC, and KP neurons

Receptors expressed in AgRP, POMC, and KP neurons based on 'KEGG BRITE' receptor categories (ko04030, ko04050, ko04054 and ko03310, and ACVR1C and INSR from ko01001 + K08873; for Figure 6b and Figure 7).

Supplementary Data 13. Orthologous genes expressed in human and mouse AgRP and POMC neurons

Transcriptome data of all recognized orthologs in human and murine hypothalamic AgRP and POMC neurons

Supplementary Data 14. Human–mouse neuropeptide orthologs in AgRP and POMC neurons

Expression patterns of neuropeptide gene orthologs in human and murine hypothalamic AgRP and POMC neurons (for Figures 8a, b).

Supplementary Data 15. Human–mouse receptor orthologs in AgRP and POMC neurons.

Expression patterns of receptor gene orthologs in human and murine hypothalamic AgRP and POMC neurons (for Figure 8c).

Supplementary Data 16. Spatial transcriptome profiling of POMC neurons in the ventrolateral and dorsomedial infundibular nucleus

Transcriptome profiles of POMC-immunoreactive neuronal populations in the ventrolateral (VL) and dorsomedial (DM) subdivisions of the human infundibular nucleus (for Figure 9).
